# Modernizing thermal discharge assessments for the 21st century

**DOI:** 10.1002/ieam.4472

**Published:** 2021-07-22

**Authors:** Lawrence W. Barnthouse, Charles C. Coutant

**Affiliations:** ^1^ LWB Environmental Services, Inc. Hamilton Ohio USA; ^2^ Coutant Aquatics Oak Ridge Tennesee USA

**Keywords:** alternative thermal‐discharge limit, balanced indigenous population (BIP), Clean Water Act §316(a)

## Abstract

A jointly prepared, interagency (US Environmental Protection Agency [USEPA] and the US Nuclear Regulatory Commission [USNRC]), §316(a) Technical Guidance Manual has been the primary guide to ecological studies of thermal discharges at power plants since 1977. It reflected contemporary ecological theory, which assumed that undisturbed populations and ecosystems possessed a balanced and relatively unchanging structure and function that could be disrupted by addition of heat from a thermal discharge. It was intended primarily to facilitate the licensing of proposed nuclear power plants and thus focused on predictive assessments. Since 1977, however, scientific and regulatory contexts of §316(a) assessments have changed. Ecologists abandoned the notion of “balance” in populations and ecosystems and now recognize that natural systems are always changing spatially and temporally. Regulatory emphasis has shifted from predictive assessments at new plants, largely based on thermal‐tolerance laboratory data, to retrospective assessments based on field data at operating plants. We suggest updates to thermal‐assessment studies based on modern ecological theory and recent thermal‐assessment practice. The concepts we outline are fully consistent with statutory language and may assist in design and implementation of study plans by applicants and their consultants, development of discharge permits by USEPA or state agencies, and reviews of assessment documents by interested public and environmental organizations. *Integr Environ Assess Manag* 2022;18:459–468. © 2021 The Authors. *Integrated Environmental Assessment and Management* published by Wiley Periodicals LLC on behalf of Society of Environmental Toxicology & Chemistry (SETAC).

## KEY POINTS

- The 1977 EPA guidance on thermal effects assessment employed an outdated definition of the concept of “balance.”
- The guidance was also directed at predictive assessments of potential effects at power plants that had not yet been built.
- We develop a revised definition of the term “balanced indigenous population” that is consistent with modern ecological theory.
- We also provide recommendations for the design of thermal effects studies at existing power plants using site‐specific ecological data.

## INTRODUCTION

Section 316(a) of the Clean Water Act (CWA) permits the owner or operator of a point‐source discharge to demonstrate that otherwise applicable effluent limitations on the thermal component of the discharge required under sections 301 or 306 of the Act (considering interaction with other pollutants) are more stringent than necessary to assure the protection and propagation of a balanced, indigenous population (BIP) of shellfish, fish, and wildlife in and on the receiving water body. Although applicable to any facility that discharges heated water, this provision has been most prominently applied to nuclear and fossil‐fueled electric power plants.

The US Environmental Protection Agency (USEPA) developed regulations implementing §316(a) that are codified at 40C.F.R. Parts 125.70–125.73, known as Subpart H. These regulations provide definitions for terms used in the CWA and identify the process and major decision criteria for determining if an alternative effluent limitation (AEL; i.e., a thermal variance from the otherwise applicable effluent limit based on adopted temperature water quality standards) may be included in a permit. In 1977, the USEPA and the US Nuclear Regulatory Commission (USNRC) issued technical guidance intended to provide permit applicants with the information needed to design, implement, and interpret studies that would satisfy the requirements of §316(a) (USEPA & USNRC, 1977). No further guidance documents have been issued since 1977, although subsequently, some states have adopted state‐specific §316(a) implementation guidance.

Although the 1977 guidance has never been updated, subsequent court decisions and administrative actions by USEPA have provided important clarifications and expansions to the guidance. Moreover, scientific understanding of the structure and dynamics of ecological systems has undercut the key operational concept underlying thermal discharge regulations: the “balanced indigenous population.” USEPA recognized early on that, rather than referring to a specific population of plants or animals, protection and propagation of the BIP really meant protection and propagation of a balanced indigenous *community* (BIC), including all of its various interacting populations. However the term is defined, since the issuance of the 1977 guidance both experience in applying the guidance and advances in the underlying ecological science have raised many questions concerning the applicability of BIP/BIC concept, specifically the meaning of the term “balance.”

Decades of ecological research and monitoring have now demonstrated that there is no constant, stable BIP/BIC in most surface waters (Coutant, 2019). Aquatic populations and communities at any location are always changing, as demonstrated wherever long‐term studies have been conducted. Many species exhibit natural abundance cycles over periods of years. If not strictly cyclic, differing year‐class successes caused by weather and biological interactions induce variability. Native fish species immigrate into new areas. Exotic species are introduced and invade new territory, which affect relative abundance of those considered “indigenous.” General warming caused by climate change allows species’ range shifts. Hydrologic events (floods, droughts) can induce aquatic life to move.

Aside from the questionable interpretations of the BIP/BIC concept, the focus of regulating thermal discharges has shifted from licensing new discharges to re‐permitting existing discharges. The 1977 guidance was devoted primarily to predictive studies at new plants, which necessarily were based on thermal model predictions and laboratory‐derived, thermal‐tolerance studies. Permit applications for existing facilities now usually include site‐specific biological monitoring data intended to demonstrate the absence of adverse effects from current facility operations. The 1977 guidance provides little information concerning the design and implementation of these retrospective assessments.

This paper (1) summarizes judicial and administrative precedents that have clarified the regulations implementing §316(a), (2) discusses current ecological understanding of aquatic community dynamics and proposes a 21st century interpretation of the BIP/BIC, and (3) suggests modernization of thermal‐discharge assessment practices to accommodate the reinterpretation of the BIP/BIC, and provide guidance designed for today's primarily retrospective assessments.

## WHAT HAS CHANGED SINCE 1977?

After publication of the 1977 guidelines, USEPA's judicial and administrative decisions or recommendations provided further clarification of the CWA and elaboration of the implementing regulations and guidance. Those that are science‐related and affect thermal‐discharge studies have been nearly universally incorporated into recent demonstrations. These changes are summarized below and discussed in greater depth in Supporting Information 1:

- clarification of the importance of site‐specific criteria (in contrast to water temperature standards that are applicable to relatively large segments of waterbodies),
- requirement to use the best information reasonably attainable (with acknowledgment of uncertainties),
- approval to base decisions on a subset of all aquatic life (representative important species [RIS]),
- requirement to evaluate sublethal or “indirect” impacts of added heat,
- requirement that the report contain a narrative summary of the study as well as technical data and technical discussion,
- approval for evaluating a localized zone wherever the affected waterbody is large (as for coastal sites),
- inclusion of habitat formers such as seagrasses as RIS,
- inclusion of both individual species and the aquatic assemblage as a whole in the data and analyses,
- necessity of focusing on the magnitude of the changes in the community as a whole and on individual species and then determining if these changes are “appreciable,”
- requirement to consider the effect of conditions that are less favorable than average (worst case) in §316(a) demonstrations,
- requirement to evaluate effects on endangered species, even if that means relaxing other considerations,
- need to consider trends in the aquatic community's history of improvement or degradation,
- need to include nearby reference areas that are not affected by the thermal discharge as a baseline for comparison,
- in waters already harmed, evaluation of whether the thermal discharge caused or contributed to the harm and will the discharge improve or further degrade conditions,
- recommended use of thermal modeling of the water body and critical temperature exceedances for RIS or the aquatic community as a reasonable basis for evaluating a proposed variance (especially for extreme conditions not actually measured during studies at the site).

After publication of the 1977 guidance, ecologists began to question the scientific basis of the concept of a BIP and to explore new ways to conceptualize the environmental impacts of stressors, including thermal discharges, such as formalizing ecological risk assessment (Barnthouse & Suter, 1984; US Environmental Protection Agency [USEPA], 1992). Finally, as mentioned, regulatory attention turned from permitting new thermal discharges to permit renewals for existing discharges. For existing facilities, the predictive evaluations described in the 1977 guidelines were now supplanted with, or supplemented by, retrospective evaluations based on site‐specific environmental monitoring data.

## A 21ST CENTURY DEFINITION OF THE “BALANCED INDIGENOUS POPULATION”

The CWA does not provide a definition of the term “balanced, indigenous population,” commonly referred to as the “BIP.” Subsequent regulations implementing the CWA, including the 1977 guidance document, interpret the term “population” to refer to a community of interacting populations (i.e., a balanced indigenous community or BIC). The guidance also provides a definition of the term “community:”

(1) “A balanced indigenous community consists of desirable species of fish, shellfish, and wildlife, including the biota at other trophic levels which are necessary as a part of the food chain or otherwise ecologically important for the maintenance of the community.”
(2) “The balanced indigenous community can include non‐native species, if (1) they are present due to major modification of the water body such as impoundments, or to watershed modification due to urban or agricultural development, (2) result from management intent, or (3) are species or communities whose value is primarily scientific or aesthetic.”
(3) “A community in general is any assemblage of populations living in a prescribed area or physical habitat; it is an organized unit to the extent that it has characteristics additional to its individual and population components, and functions as a unit through coupled metabolic transformations.”

This language reflected the state‐of‐the science of ecological theory in the early 1970s, which assumed that undisturbed populations and ecosystems possessed a balanced and relatively unchanging structure and function, which could be disrupted by the addition of heat from a thermal discharge. The phrase “…it is an organized unit to the extent that it has characteristics additional to its individual and population components, and functions as a unit through coupled metabolic transformations” illustrates the problematic nature of the term “BIP” (or “BIC”) from the perspective of modern ecological theory. Many of today's ecologists deny that any such entity as a “balanced” community (indigenous or otherwise) even exists, and even those who do accept the concept of balance apply it in a way that is very different from the way it was understood 50 years ago. As discussed below, ecological theory recognizes that natural systems are always changing and current methods for studying populations and ecosystems account for these changes.

### History of the balance concept

According to Egerton (1973), the concept of a “balance of nature” can be traced back to ancient Greece, where it had theological and philosophical roots. Abundances of different types of animals remain bounded within limits because the gods designed them to be able to coexist. Writers in the 17th and 18th centuries more fully developed the concept of the balance of nature, that is, the relative constancy of numbers of different kinds of animals as being a great wheel of life and death analogous to the rising and setting of the sun, ordained and maintained by God.

In the 20th century, a competing approach that emphasized the role of abiotic environmental fluctuations in controlling population numbers developed (Andrewartha & Birch, 1954; Elton, 1930). Nonetheless, the idea of a balanced community maintained by biotic control of populations persisted and was most forcefully expressed in the influential textbook *Principles of Animal Ecology* (Allee et al., 1949). The theoretical literature of the mid‐20th century, as exemplified by Lewontin (1969) and May (1973), also emphasized a balance maintained by biotic interactions.

The definitions provided in the regulations and guidance implementing §316(a) reflect the balance of nature concept of Allee et al. (1949) and the mathematical foundation provided by mid‐century theoreticians. However, the balance concept subsequently came under strong attack. Ehrlich and Birch (1967) contended that the common arguments for the existence of a balance are all fallacious and inconsistent with the observed dynamics of actual populations and communities.

In another treatment of the same issues, Connell and Sousa (1983) discussed several concepts related to the stability of populations and communities, from both theoretical and empirical perspectives. Reviewing available empirical studies, the authors found that none of these concepts had ever been shown to be applicable to real populations or communities. These authors investigated studies of fluctuations in natural ecosystems in search of evidence of the existence of stability, as defined in the theoretical literature. They found none and argued that classical ideas of stability are inapplicable to real ecological systems. Large fluctuations in the relative abundance of different species are normal and do not necessarily indicate instability or an unhealthy state.

Simplistic notions concerning the balance of nature were abandoned by most ecologists following these and other similar critiques. Yet, both natural events and anthropogenic disturbances such as severe overfishing can clearly cause irreversible changes (imbalances) in populations and communities. Ecologists began developing a theoretical framework that could account for both normal variability and catastrophic change.

Holling (1973) introduced a distinction between stability, defined as the ability of a community to return to equilibrium if disturbed, and “resilience,” defined as the ability of a community to remain within a “domain of attraction” despite frequent disturbances and high variability. Since Holling's (1973) paper, numerous theoretical and empirical studies have elaborated on the themes of resilience and catastrophic change in biological systems. According to Scheffer et al. (2001), sudden shifts in ecosystem structure are often preceded by periods of slow, continuous change. These continuous changes, which could be attributed to nutrient loading, overfishing, climate change, or other gradual long‐term environmental processes, reduce the resilience of ecosystems, making them more vulnerable to sudden structural shifts caused by random environmental events. These shifts in structure, such as loss of transparency and vegetation in shallow lakes subject to human‐induced eutrophication (Scheffer et al., 1993), or shifts in marine food‐web structure from benthic to pelagic fish production (Daskalov, 2002; Fogarty & Murawski, 1998; Steele, 1998) can be very difficult to reverse. Scheffer et al. (2001) cited examples involving lakes, coral reefs, woodlands, deserts, and oceans.

While the above studies focused on processes that can cause catastrophic changes in populations and communities, others have focused on processes that work to stabilize these systems. Much of this literature focuses on the importance of spatial heterogeneity among habitats (Levin, 1992). A regional environment can be conceived of as a mosaic of habitat patches that are constantly changing, becoming either more or less suitable for particular species or communities. Species can be eliminated periodically from any particular patch of habitat yet persist indefinitely provided that suitable habitat patches are available elsewhere and individuals are able to disperse between patches. Hence, at the level of a region containing a variety of shifting habitats, a community can appear “balanced,” although at smaller scales it can often be unbalanced. Brennan et al. (2019) recently provided an example of this type of balance involving chinook and sockeye salmon in the Nushagak River watershed, Alaska. These authors found that subwatersheds within the Nushagak River system varied substantially from year‐to‐year with respect to salmon production, yet annual returns from the river system remained relatively constant. Brennan et al. (2019) concluded that maintaining the heterogeneity and connectivity of the watershed is critical for ensuring the resilience of salmon production.

### A 21st century definition of the BIP/BIC

Referring to the definition of a community provided in the 1977 guidance, if “prescribed area or physical habitat” refers to a specific location such as the vicinity of a thermal discharge, then “balance” cannot mean a static assemblage of populations with relative abundances that are stable over long periods, because random environmental events such as floods, droughts, and storms, and long‐term processes such as climate change will inevitably introduce variability and the possibility of local extinction with or without a thermal discharge. Current understanding of ecosystem structure and dynamics seems to require that the 1970s concept of the BIP/BIC be expanded to include consideration of spatial extent, connectivity, and resilience. The following expanded definition (expansion italicized) of a BIC is one approach to satisfying these requirements:

“A balanced indigenous community consists of desirable species of fish, shellfish, and wildlife, including the biota at other trophic levels that are necessary as a part of the food chain or otherwise ecologically important to the maintenance of the community.”
“The balanced indigenous community can include non‐native species, if (1) they are present due to major modification of the water body such as impoundments, or to watershed modification due to urban or agricultural development, (2) result from management intent, or (3) are species or communities whose value is primarily scientific or aesthetic.”
“*A community in general is any assemblage of populations living in a prescribed area or physical habitat that is (1) large enough to contain self‐sustaining populations of all of the species necessary to maintenance of the community, or (2) connected by migration corridors to similar communities that together are sufficient to sustain community structure and function. A balanced community is resilient in the face of environmental variability to the extent that key functions are maintained in spite of fluctuations in environmental conditions and species abundances. Appreciable harm to the BIC could occur if a thermal discharge (1) affects an area large enough to threaten the sustainability of key ecosystem components considering temporal patterns of exposure, (2) leads to blockage of migration corridors essential to maintaining watershed connectivity, or (3) increases the likelihood that the community will shift to an alternative, less desirable, state.*”

The remainder of this paper discusses changes in thermal discharge assessment practices that account both for the new interpretation of the BIP/BIC and for the shift in regulatory objectives from initial licensing of new plants to re‐permitting of existing plants. Case studies summarized as part of this discussion demonstrate that some recent §316(a) studies have already incorporated elements of our new definition.

## MODERNIZING §316(A) STUDIES

These recommendations are based on a review of 40 years of §316(a) implementation and a thorough understanding of modern ecological principles. Most of the recommendations provided here are modest updates to concepts already present in the 1977 guidance, and all are consistent with statutory language. Even without any formal changes in regulation or guidance, these recommendations could still be used by §316(a) applicants in the preparation of their demonstrations. In fact, as discussed below, some of these recommendations have already been applied in §316(a) assessments that were either approved by regulatory agencies or performed by USEPA itself.

Today's thermal‐discharge studies should consider the changing landscape for how much information is necessary to obtain a variance. In most cases, today's answer is “more” rather than “less.” The amount of information “reasonably attainable” has increased markedly with increasing numbers of thermal‐discharge studies, temperature‐related ecological studies, and ecological studies of receiving waters unrelated to thermal discharges. Federal and state agencies have conducted monitoring studies of many water bodies that receive thermal discharges, including estuaries, the Great Lakes, and large rivers (Mississippi, Missouri, Ohio). Multimetric indices of fish community structure are now routinely used to assess the biological integrity (another term for “balance”) of communities present in streams, rivers, and lakes throughout the United States (Karr & Chu, 1999). Experiments in thermal tolerance, physiology, and behavior of many relevant species can be found in the published primary literature, topical books, and summary reports. USEPA has produced documents that contain relevant information, such as for development of water temperature standards. The breadth and depth of information attainable from well‐designed and executed field studies has increased greatly through advances in field‐study techniques, sampling‐station siting, taxonomic identifications, computer‐formatted and searchable databases, and computational methods.

With the increased amount of “attainable” or available information has come expanded expectations by USEPA and the delegated states for thorough documentation of support for a proposed AEL for existing thermal discharges. Many old §316(a) demonstrations and their resultant permit limitations have been called into question and regulators have required up‐to‐date demonstration studies and documents. The demonstrations most likely to be accepted are those with ample documentation from field study of the site, corroborating third‐party studies, and species‐specific information from the published literature.

### Combined Type I and Type II/III demonstrations

The 1977 guidance categorized demonstrations as either Predictive or Non‐predictive (absence of prior harm). Predictive assessments, that is, environmental assessments based on information from the environmental and biological literature rather than from field studies of operating thermal discharges, were termed “Type II” and “Type III” demonstrations. Demonstrations based on retrospective analysis of field data were designated as “Type I” demonstrations. The guidance allowed applicants to choose between submitting a Type I or a Type II/III demonstration.

The distinction between Type I and Type II/III demonstrations is largely outmoded today for existing discharges because field data are now required for nearly all §316(a) demonstrations. This does not mean, however, that predictive data are no longer relevant. Predictive analyses of the types discussed in the 1977 guidance are not an alternative to field studies but rather a valuable addition to those studies. This is in part because the field studies for a §316(a) demonstration are rarely conducted when ambient or discharge temperatures are highest. Thermal modeling can, however, simulate those temperatures and other thermal regimes in the receiving water that reflect the historical record of the water body and thermal releases. Available laboratory and life‐history data on aquatic species or assemblages can be compared with the results of this thermal modeling.

Adding predictive assessment to the retrospective demonstration would, in USEPA's words, use the best information reasonably attainable for estimating harm (or lack thereof). The information includes basic life‐cycle thermal requirements of key species tested in the laboratory as well as field data on occupied temperatures from studies elsewhere published in the literature.

Conceptually, combined retrospective and predictive assessments of the impacts of a thermal discharge on aquatic life can be described in terms of three components borrowed from USEPA's generalized framework for ecological risk assessment (USEPA, 1992, 1998): exposure analysis, effects analysis, and risk characterization.

#### Exposure analysis

Exposure analysis is the tracking of temperatures an organism would experience over time, for example a fish (like a juvenile downstream‐migrating salmon) passing through a thermal plume, or contact of the plume with critical, fixed spawning habitat. Both temperature monitoring studies and thermal plume modeling are needed for an adequate thermal exposure analysis.

The large number of thermal‐discharge studies that have been carried out since the CWA of 1972 (and even earlier) have clarified what temperatures need to be measured routinely to understand spatial and temporal patterns. The temperatures fall into three general categories: (1) ambient (with no influence of the thermal discharge) including at reference (control) study sites, (2) thermally affected (thermal plume), and (3) areas in the trajectory of the thermal plume where the temperature is expected to return to near ambient. Long‐term records are especially valuable for demonstrating the norms and extremes. Many of these data already exist and represent valuable data resources for modern §316(a) demonstrations. In addition to being needed to permit calibration of thermal plume models, observational data address a the principal limitation of advanced thermal plume models: restriction of the analysis to a relatively small number of highly specific exposure scenarios, with limited ability to characterize the frequency of occurrence of various exposure conditions. However, temperature monitoring is usually limited to a few fixed stations. For this reason, the fine‐scale spatial distributions of temperatures at various locations within a plume, and also variations in plume locations as functions of environmental conditions, cannot be accurately quantified without models.

From a predictive perspective, state‐of‐the‐art, three‐dimensional modeling of the mixing zone can estimate water temperatures from the discharge point to nearly complete mixing with the ambient water body. The state‐of‐the‐art of modeling temperatures in thermal discharges has changed markedly since the 1970s. The changes have involved both improvement in models and increases in model availability. Three‐dimensional modeling of the thermal discharge's mixing zone can estimate water temperatures from the discharge point to nearly complete mixing with the ambient water body. Commercially available computer models (Flow 3D, RMA‐10, CORMIX, or similar) can be applied by consulting firms to the site under varying conditions of meteorology, flow (river, tidal, or long‐shore), water temperature, bathymetry, and plant operations. Delineation of the thermal plume and the ability to model seasonal, extreme, and statistical (e.g., 90% occurrence) scenarios assist in selection of biological sampling locations and quantification of temperatures and exposure durations.

Determining realistic thermal exposures for mobile organisms, such as subadult or adult fish, can be a challenge, however. Because USEPA has emphasized maintaining a zone of passage past a thermal plume, one can estimate the time and temperature pattern for actively swimming through the plume. For other less directed movements, such as avoidance of the warmest waters, temperature preference data are useful for estimating temperatures that fish will avoid (Coutant, 1975, 1977).

#### Effects analysis

Modern §316(a) demonstrations for existing facilities can use a combination of field data on aquatic populations and communities and predictive data derived from laboratory‐derived, thermal‐tolerance studies.

Three general types of field studies have been conducted in ecosystems affected by thermal discharges: before–after comparisons, reference area comparisons, and before‐after control‐impact (BACI) analyses. The most commonly available studies are before–after comparisons, as differentiated below. In some cases, the “after” data have been collected 10 years or more following the “before” data, for example, when a regulatory authority has required that a new study of the aquatic communities in the zones affected by the temperature elevations from the thermal discharge be performed in connection with a permit renewal. In other cases, annual field studies may have been performed, permitting long‐term trends in population abundance and community metrics to be analyzed. The fact that populations and communities are in a constant state of flux irrespective of thermal discharges complicates the interpretation of before–after data and trend analyses. BACI analyses avoid many of these complications by conducting Before and After studies at Control (or reference) sites as well as the Impacted site. The BACI approach provides a measure of change in the affected zones relative to simultaneous changes in the control or reference area(s). This approach works only when the “control” and the “impact” sites have been subject to similar sequences of background environmental influences (Stewart‐Oaten et al., 1986; Thomas et al., 1978). The BACI approach requires careful consideration of the most appropriate statistical analyses.

BACI studies are possible only if they have been designed into the original monitoring plan for a facility, before the initiation of the thermal discharge. Reference area comparisons are likely to be the best types of studies at facilities where no field studies were performed before starting the heat addition or the earlier studies were many years ago. Reference areas should be as similar as possible to the plume‐affected areas (e.g., similar habitat characteristics) and should be sampled during the same seasons and using the same sampling techniques. If possible, multiple reference areas should be sampled. Where they exist, results of third‐party studies should be examined and compared with results obtained from facility‐specific monitoring.

Although field data may suggest no harm to exposed populations and communities, the case is much stronger when appropriate laboratory data are included. This is especially important for making the case that certain biotic categories are “low potential impact” in the environment of the thermal discharge. Historically, when the effects of future power plants were the main concern, these laboratory data were most of the available information. Now, they augment the field data, especially for environmental and thermal‐discharge conditions not actually studied. They also can be used in evaluating specific, representative thermal exposures not amenable to the usual methods of field collections.

Thermal endpoints generally include values such as upper temperature tolerance limits for survival (generally 50% survival of a test group), growth rates at a range of temperatures (including the optimum temperature), preferred and avoidance temperatures, and embryo development rates. For many species, critical temperatures vary by life stage. Early laboratory studies generally tested the survival of test organisms at specific temperatures, recording the duration of time required for 50% of the endpoint. The endpoint for some studies was equilibrium loss, considered an ecological survival endpoint caused by likely predation on a disoriented animal (Coutant, 1970, 1973; Coutant & Dean, 1972). When evaluating the applicability of any laboratory‐derived, thermal‐tolerance study, the mechanics of how the study was conducted (e.g., Critical Thermal Maximum [CTM] vs. Upper Incipient Lethal Temperature [UILT] vs. “slow heating rate”) should be considered.

The capability to simulate biological effects of fluctuating temperatures over time is especially important. Predictions based on constant‐temperature laboratory data have been available for thermal‐discharge analyses since the 1970s. For example, the ability to estimate the survival and development rate of incubating fish eggs under fluctuating temperatures using exposure degree days is well known and regularly applied to fish‐hatchery practice and environmental analyses (Fry, 1971; Paloheimo & Dickie, 1966). Also, the computational ability to estimate physiological survival or equilibrium loss (“ecological death”) of fish passing through thermal plumes (or intermittent exposures of sessile organisms by fluctuating plume locations) based on constant‐temperature laboratory data has been described (Coutant, 1972; Jaske et al., 1969; National Academy of Sciences and National Academy of Engineering, Environmental Studies Board, Committee on Water Quality Criteria [NAS/NAE], 1973). Numerous less quantitative studies have revealed that intermittent exposures above the incipient lethal temperature are cumulative. In addition to these historical approaches, there remains opportunity to apply more recent developments in modeling thermal effects under fluctuating temperature conditions, including evidence of some recovery in the period between high‐temperature exposures (Bevelheimer & Bennett, 2000).

#### Risk characterization

“Risk characterization” as discussed by Suter (2007) is a process that integrates information on exposures and effects of stressors and interprets the results in a form that supports reasoned environmental decisions. Modern §316(a) demonstrations will often be based on multiple lines of evidence, including both laboratory and model‐derived predictions and field observations.

For example, with a temperature‐time curve derived from a thermal plume model in hand, laboratory‐derived resistance times at constant temperatures can be used to predict the survival (or not) of the tested organism using available methods (Coutant, 1972; NAS/NAE, 1973). That is, whether or not they accumulate a lethal dose of high temperatures during their exposure. Similarly, the likely temperatures and timing of intermittent exposures of an important habitat by a tidally fluctuating thermal plume can be characterized by the model and the biological effects estimated.

For some facilities, such predictive analyses could be the only type of evidence concerning the likelihood that a discharge will cause harm to the BIP/BIC. However, for most existing facilities field data on the abundance and distribution of the RIS, and on the characteristics of the aquatic community as a whole, are likely to be available. In all four of the case studies documented in Supplement 2 and summarized below, multiple sources of field data were available, both from owner‐sponsored monitoring and from third‐party studies. Field data provide direct evidence of whether or not a thermal discharge caused harm to the BIP/BIC in the receiving water body. Do long‐term trends reveal declines in the abundance of thermally sensitive RIS that cannot be explained by other factors? Is the fish community exposed to the discharge significantly different from the communities present in reference areas? Although a BIP/BIC is not required in the immediate zone of the discharge (often regulated as a mixing zone), the zone may influence the community outside that zone, something that, if adverse, is to be avoided.

As noted elsewhere in this paper, interpretation of field observations is complicated by factors such as natural environmental variability, unrecognized differences between exposure and reference sites, and long‐term environmental trends independent of thermal discharges. For this reason, predictive analyses are a valuable adjunct to field studies. Conclusions concerning the presence or absence of harm are especially strong if predictive and retrospective analyses are consistent. For example, if laboratory effects data and thermal modeling predict that no adverse effects should occur, and field data demonstrate that none have been observed, then it can confidently be concluded that the discharge has caused no appreciable harm to the BIP/BIC. However, different lines of evidence may also conflict. Modeling may predict that effects on one or more RIS should be observed, but field data do not indicate adverse changes in RIS populations; or predictive models may predict no effects although adverse changes in one or more RIS populations are observed. In such cases, a “weight‐of‐evidence” procedure can be used to evaluate the reliability of the various lines of evidence available and develop the conclusion that is best supported by the available data.

The wide variety of circumstances and types of data available for site‐specific §316(a) demonstrations preclude us from providing a specific weight‐of‐evidence procedure in this paper. Many such procedures have been used in ecological risk assessments for pollutant discharges and contaminated sites; Suter (2007, chap. 32) provides a detailed discussion of the topic.

#### Demonstration format: Biothermal assessment and master rationale

The published literature on ecological risk assessment places major emphasis on communication of risk assessment conclusions to decision makers and the public (Suter, 2007; USEPA, 1998). Risk characterization documents are expected to be “clear, transparent, reasonable, and consistent” (USEPA, 1998). As noted by Suter (2007), there are often conflicts between these characteristics. Transparency requires that data and analytical methods be documented in sufficient detail to support the assessment's conclusions. Clarity, on the other hand, requires that assessment documents not be excessively long or complex. The Master Rationale described in the 1977 draft guidance for §316(a) demonstrations was USEPA's attempt to resolve this conflict. With some modifications, the Master Rationale can still serve this role.

As described in the guidance (p. 52):

“The Master Rationales of the demonstration should summarize the key findings in a concise manner and should form a convincing argument that the balanced, indigenous community will be protected. The rationale should include a summary of an ‘overall picture’ of the ecosystem as projected by the six Biotic Category Rationales, the resource zones impacted, and a summary of why the information in the rationales, along with the predictions in the RIS Rationale, the engineering and hydrological data, and other key facts, suggest that the balanced indigenous community will be protected.”

The basic concept of a master rationale has not changed since the 1977 guidance. Every demonstration is intended to make the quasi‐legal case that the document shows factually that there is no prior appreciable harm from the existing thermal discharge. It is the main place where the information from site characteristics (e.g., habitats and important resource zones), thermal plume modeling, literature on the biological requirements of RIS, historical hydrological data, engineering design of the discharge, field‐study collections, and other relevant facts are brought together to support a conclusion regarding the protection and propagation of the BIC. It summarizes factual evidence of the lack of prior appreciable harm (as opposed to an opinion‐based lack of evidence of harm). The only change required to apply the concept to an integrated predictive and retrospective assessment would be to add discussions of field‐based lines of evidence and weight‐of‐evidence evaluations to the existing guidance on presentation of thermal tolerance and other relevant data.

The purpose of a §316(a) demonstration is not only to show lack of appreciable harm, but to make the case for an AEL that meets the criteria for a BIP/BIC. That is, an alternative to the receiving body's applicable water quality standards or the imposition of closed‐cycle cooling. The alternative is really the endpoint of the demonstration that has to bridge ecology (messy) and compliance monitoring (simple and easily measured). It is commonly based on the recent operational history of the facility, assuming no prior appreciable harm has been demonstrated. The delineation of an AEL can take many forms, and these are typically negotiated between the applicant and the regulatory agency. In some cases, however, the administrator selects an alternative different from that proposed by the applicant (e.g., Brayton Point Station). Although a proposed AEL might be deferred until negotiations over a draft permit, it is preferable to present the case for the alternative in the document and its Master Rationale.

## FOUR RELEVANT EXAMPLES

Here, we summarize four rather different examples of recent §316(a) assessments. Detailed discussions of all four are provided in Supporting Information 2. Brayton Point Station was on an East Coast estuary, Cardinal station is on the impounded Ohio River, the Labadie Energy Center is on the free flowing but channelized lower Missouri River, and the Evergreen Canton Mill is on a small river in the Appalachian Mountains. All four demonstrations relied on similar types of data: historical and current surveys of fish populations, other community components, and community dynamics in near‐field and far‐field locations. However, the approaches to organizing and presenting the information were substantially different.

The Brayton Point Station assessment was organized around the 1977 federal guidance, which provides minimal guidance on retrospective (Type I) assessments. USEPA applied the biotic categories and decision criteria developed for predictive (Type II) assessments but using empirical data rather than literature‐derived predictions. The Cardinal assessment, in contrast, was organized around the 1978 Ohio Environmental Protection Agency (OEPA) guidance (OEPA, 1978), which provides a concrete list of characteristics for using empirical data to determine whether or not harm to the BIP/BIC has occurred. The Labadie assessment generally followed the 1977 guidance for predictive Type II analyses while conducting a Type I field study guided by consideration of thermal modeling in study design and inclusion of RIS. The study and its conclusions regarding harm were guided by a checklist of criteria from the USEPA regulations and accumulated administrative decisions and advice (similar to the OEPA's list of characteristics). The Canton Mill demonstration, in addition to including both thermal modeling and field studies at multiple reference locations, accounted for improvements in fish and benthic community quality related to reduced pollutant discharges under successive variances over a period of approximately 30 years.

It is reasonable to have regional differences in how §316(a) assessments are conducted, because most decisions regarding NPDES permitting, including thermal discharges, have been delegated by USEPA to the states. However, as long as the best available information is obtained, interpreted according to scientifically accepted procedures, and presented in a clear and transparent manner, there is no need to adhere to a rigidly prescribed demonstration format, provided that all regulatory requirements are satisfied. What is critical to note here is that all four of these assessments integrated retrospective and predictive approaches, and all four employed interpretations of the BIP/BIC that are consistent with the modernized definition developed in this paper.

## DISCUSSION

This review was motivated by the problematic use of the term “balanced indigenous population” in §316(a) of the CWA and the focus of the 1977 draft technical guidance manual on predictive assessments rather than actual field data. Interestingly, moderate updates rather than drastic revisions appear to be appropriate. We briefly discuss the main aspects of guidance modernization that could be considered.

Ecologists have long recognized that aquatic communities are not in a stable balance of species and their relative numbers, even in the absence of anthropogenic stressors. From a scientific perspective, maintaining a “balanced indigenous population” or “balanced indigenous community” is an unattainable goal as long as the term “balance” is interpreted to mean an absence of change. Because these terms are founded in the CWA legislation and are likely immutable, this paper focuses on developing revised interpretations that are still consistent with statutory language.

Both the ecological literature and USEPA Region 1's analysis of the Brayton Point Station indicate that “balance” can move from a static ecological condition to a more modern, comparative one. “Balance” at a thermally affected site is a relative measure that is viewed in comparison to reference locations (preferably more than one) with similar habitats and history other than added heat. This interpretation is consistent with the statutory language of §316(a) and is also consistent with approaches used to design ecological risk assessments at contaminated sites (Suter, 2007).

Easy‐to‐follow decision criteria could improve guidance for demonstrations. Such criteria, in checklist form, could help both study planners and regulators focus the assessments on pertinent local environmental issues (Coutant, 2019). History shows that this may not happen if the demonstration is focused on just the four main topics of the regulations. Such lists are ideally tailored to the specific location, where some criteria on the list are not relevant and others of local concern need to be added. Where state agencies such as the Ohio USEPA have developed criteria, facilities in these states must follow these criteria. Because the approach to §316(a) assessment varies among states, it is important to develop study plans that reflect the state‐specific approach for the state where the study will be conducted.

Cumulative impacts are important, as exemplified by the Brayton Point Station. Demonstrations of no prior appreciable harm require evaluation of the “cumulative impact of its thermal discharge together with all other significant impacts on the species affected” [CFR 125.73(a)], and should go beyond “interaction of such thermal component with other pollutants” [CFR 125.70]. Cumulative ecosystem effects at Brayton Point Station were viewed as total ecosystem responses (e.g., shifts in community composition and ecosystem processes from that seen in reference areas) as well as the usual evaluation of interactions of added heat with other stressors (e.g., dissolved oxygen, toxicants, overfishing, predators). This broader perspective was key to that decision.

Repeated field studies at a site, such as for discharge permit renewals, are valuable for identifying trends in species success and ecosystem conditions, as exemplified by the Canton Mill case study. A series of thermal‐discharge evaluations is especially valuable if concurrent with water quality improvements. The series could identify trends leading to an improved BIP/BIC despite a thermal discharge with AELs (variances). Conversely, trends may be negative, as was demonstrated in multiple studies of ecosystem components in the Brayton Point Station case, leading to different limitations than proposed or rejection of the proposed variance entirely.

We favor elimination of the distinction between Type I (retrospective) and Type II/III (predictive) assessments in favor of integrated assessments that incorporate both retrospective and predictive elements. This integration would align §316(a) assessments with ecological risk assessment principles developed since 1977 that are applicable to many environmental regulatory issues (Suter, 2007; USEPA, 1998). Many recent demonstrations already include predictive modeling, field data, and estimated effects of atypical temperatures on key species (RIS). Eliminating the distinction between Type I and Type II/III assessments would make the guidance consistent with practices already followed by applicants and accepted by USEPA and state agencies.

In recent years some states have issued updated §316(a) implementation guidance documents reflecting 21st century assessment methods and have used these methods to address the protectiveness of previously approved §316(a) variances (e.g., Indiana Department of Environmental Management [IDEM], 2015). Our four diverse case studies integrated retrospective and predictive approaches, and all four employed interpretations of the BIP/BIC that are consistent with the modernized definition developed here.

The 1977 draft §316(a) guidance was never finalized, and it seems unlikely that USEPA will revise that document in the foreseeable future. However, the concepts in this report could still be adopted by permit applicants when designing and executing new §316(a) studies. We assume applicants will continue to seek permit renewals from state agencies or USEPA for existing discharges. Not all applicants will be required to perform new field studies, but those applicants that must perform new studies can use this report to support development of §316(a) demonstrations that satisfy the requirements of permitting authorities.

## CONFLICT OF INTEREST

The authors declare that there are no conflicts of interest.

## DISCLAIMER

Although this research was funded by the Electric Power Research Institute, the views expressed are those of the authors and do not reflect official positions of the sponsoring organization. The peer review for this article was managed by the Editorial Board without the involvement of L. W. Barnthouse.

## Supporting information

This article includes online‐only Supporting Information.

**SUPPLEMENT 1.** Summarizes legal and regulatory decisions made since 1977 that affect the conduct and interpretation of thermal discharge studies.

**SUPPLEMENT 2.** Documents case studies of four recent thermal discharge assessments that illustrate advancements in the field.

## Data Availability

No original data were collected as part of this research. Details concerning regulatory developments and case studies discussed in this paper are provided in the Supporting Information.
