# Supplementary material for: Modernizing thermal discharge assessments for the 21st century
Source: Integr Environ Assess Manag. 2021 Jul 22;18(2):459–68. doi: 10.1002/ieam.4472 (PMC9291154; doi:10.1002/ieam.4472)
Supplement: Supplementary file 1 — SUPPLEMENT 1. Summarizes legal and regulatory decisions made since 1977 that affect the conduct and interpretation of thermal discharge studies. [file IEAM-18-459-s001.docx]

# Supplement 1: Judicial and Administrative Actions Affecting Interpretation of the Clean Water Act

This supplement reviews litigation and administrative decisions involving § 316(a) of the CWA since preparation of the 1977 guidelines for BIP/BIC demonstrations. It is derived from notes taken from summaries prepared by legal staffs in support of actual demonstrations. The authors of this report are not legal professionals and do not have the primary legal documents except for that developed for Brayton Point Station (see below). The sections that follow emphasize and explain decisions that, from the perspective of a biologist, have most affected study plans and decision criteria for determining if there has been “prior appreciable harm” to the aquatic biota at an existing thermal discharge for which a BIP/BIC is to be demonstrated.

Three sources of precedents were used by the original legal teams: (1) federal court decisions; (2) EPA administrative decisions, several of which are based upon adjudicatory hearings; and (3) EPA Office of General Counsel opinions. Most decisions that we describe involved contested issues, for which the authors of the original summaries could find complete administrative records.

Major Court Decisions

The Fourth Circuit Court concluded, in Appalachian Power Co. v. Train (1976), that compliance with existing water quality standards did not automatically satisfy the requirement of § 316(a). The section requires consideration of site-specific criteria, while water quality standards are applicable to relatively large segments of water bodies.

Major Administrative Decisions

The EPA Administrator provided several important clarifications of the regulations in the course of reviewing contested proceedings concerning discharge permits at power plants.

Pilgrim

The EPA Administrator made several precedent-setting conclusions regarding the oceanside Pilgrim Power Plant (1977).

The applicant must provide the “best information reasonably available.” The decision went on to point out that the applicant must present all relevant and reasonably obtainable data, account for any significant deficiencies, utilize available predictive methodologies effectively, and provide a reasonable basis for evaluating biological impacts. If “substantial uncertainty” exists as to the extent of harm due to insufficient information, then the applicant has failed to demonstrate that less stringent standards would protect the biota.

The Administrator also concluded that an applicant may employ one of three different methods to show that less stringent limitations will meet the § 316(a) requirements: (1) absence of prior appreciable harm; (2) demonstration that Representative Important Species (RIS) are protected; and (3) submission of relevant biological, engineering, and other pertinent data.

The administrator also presented the first list of the **major adverse impacts** to be considered, which include:

- a decrease in abundance of threatened or endangered species;
- an increase in abundance of nuisance species;
- a decrease in abundance of indigenous species;
- damage to critical aquatic organisms, such as important elements of the food chain or damage to basic ecosystem processes;
- a change in population composition (presumably meaning community);
- a decrease in commercial or sport fisheries.

The Administrator’s decision further indicated that all adverse impacts on individual species, including sublethal and “indirect” impacts of the thermal discharge must be considered. These include adverse impacts on early life stages that alter the adult population, increases in predator species, cold shock and gas-bubble disease.

Further, entrainment and impingement impacts on spawning and nursery areas must be considered in a § 316(a) demonstration along with the strictly thermal impacts. The decision also launched into obtaining a § 316(b) decision, noting that adverse impact needs to be minimized, not eliminated.

Seabrook

The Administrator made several important decisions in granting a contested variance for the Public Service Company of New Hampshire’s oceanside Seabrook Nuclear Power Plant or Seabrook Station (1977).

The decision indicates that § 316(a) requires that the applicant “demonstrate” entitlement to a variance. Hence, the burden of proof for § 316(a) rests with the applicant. The applicant must provide an “interpretive, comprehensive, narrative summary of the demonstration.” [underline in the original] The information needs to be adequate to provide “an evidentiary showing needed to make a reasoned decision.”

The information presented by the applicant does not necessitate data for thermal-discharge effects on the entire ecosystem. Rather, overall effects can be inferred from studies on selected Representative Important Species (RIS). Data on the entire RIS need not be supplied if inferences can be made from a selected few. The decision reiterated (in somewhat different words) the previous decision that the standard of evidence is “the best information reasonably obtainable.”

The decision indicated what constitutes receiving waters under § 316(a) when there are no obvious physical boundaries (such as coastal waters). “[T]he portion chosen is necessarily arbitrary to some extent” and it may be necessary to select that portion of coastal waters “where human use or enjoyment of the marine resources may be affected.”

Brunswick

The Administrator clarified the notion of adverse impacts in the contested permitting of Carolina Power and Light Company’s Brunswick Steam Plant on the Cape Fear River near Wilmington, North Carolina (1977). While upholding the prior decision that § 316(a) analyses must include entrainment and impingement impacts, the decision held “adverse” to the more stringent standard of “harmful” but not “irreversible.”

Anclote

The ecological importance and relevance for § 316(a) demonstrations of habitat forming sea grasses was stressed in permitting of Florida Power and Light’s Anclote Plant at the Anclote River in southwestern Florida (1978). The Administrator considered the demonstration’s omission of these habitat formers in his rejection of the demonstration. EPA stated that an analysis of seagrasses would be “vital information” for the demonstration since the grasses are the major primary producers in the area as well as habitat formers for animal life essential for maintenance of the required balance.

Wabash River and Cayuga

Several key precedents were established by the long-running, contested permitting of Public Service Company of Indiana’s Wabash River and Cayuga generating stations on the Wabash River, Indiana (1979).

An initial opinion by the EPA Regional Administrator stated that both plants’ thermal discharges were in compliance with § 316(a). He stated that “although appreciable harm to the balanced indigenous community of the Wabash River has been caused by the subject discharges…those discharges have been demonstrated not to preclude the protection and propagation of the [BIP].” The decision was remanded after objection by the EPA Administrator and the state. The initial decision was clarified prior to issuance of permits by the state in 1985.

The definition of balanced, indigenous population was clarified to include both individual species and the naturally occurring assemblage of organisms (i.e., the biological community).

Furthermore, “…[In] attempting to judge whether the effects of a particular thermal discharge are causing the ecosystem to become unbalanced, it is necessary to focus on the magnitude of the changes in the community as a whole and in individual species” and then determine if these changes are “appreciable.” Although the overall number of fish in the Wabash River was unaffected, some species were virtually eliminated from the power plant sites. The Administrator found that “such shifts [in populations of individual species] are at war with the notion of ‘restoring’ and ‘maintaining’ the biological integrity of the nation’s waters” as required by the Clean Water Act. However, a minimal reduction in the population of a particular species in **a** localized area was found to be acceptable, provided that the species continues to flourish in the regional area occupied by its populations.

In addition, the Administrator concluded that the effect of conditions which are less favorable than average [worst case] must be taken into consideration in § 316(a) demonstrations. The hydrological seven-day, one-in-ten-year-low-flow (“7Q10”) was used as an example.

The Administrator indicated that the “decision to grant or deny a request for less stringent thermal limitations under § 316(a) hinges solely on proof of the biological effects of the discharges.” Economic considerations occur elsewhere in setting original thermal limitations.

Regarding inclusion of entrainment and impingement, the Administrator concluded that while these impacts should be considered as part of “all relevant stresses,” the environmental impact at the intake need not be independently measured (i.e., entrainment and impingement at the plant need not be measured). It is only necessary to show that resident populations of important biota have not declined as a consequence of plant operation.

A major decision allowed power companies to avoid repeating biological studies for many years after acceptance of an initial demonstration of no appreciable harm (e.g., on the required periodic renewal of an NPDES permit). The Administrator opined that if a plant is already in operation, and no prior appreciable harm can be shown, then it may be presumed that there will be no appreciable harm in the future. Once a utility has provided facts establishing no prior appreciable harm, the burden is on the regulator and other opposing parties to rebut the presumption.

This decision is no longer being honored by EPA or state regulators. Since about the late 1980s, regulatory authorities have required updated demonstrations during the NPDES permit renewal process.

Indian River and Cape Canaveral

The importance of endangered species in § 316(a) decisions was evident in EPA’s joint evaluation of two power plants – Orlando Utilities Commission’s Indian River Plant and Florida Power and Light Company’s Cape Canaveral Plant -- on the Cape Canaveral Pool, Florida (1983). Although EPA found “significant” adverse biological impacts on a large portion of the pool, the agency concluded that the BIP was not “endangered” when all factors were considered. One important factor was the presence of the endangered manatee, which depends on the warm thermal discharges for survival in winter. This decision indicates that benefits versus risks (harm) need to be considered carefully when endangered species are involved, and the usual measures of appreciable harm may not apply.

Major Decisions by the EPA General Counsel

In 1977, the EPA General Counsel clarified the distinctions between § 316(a) and § 316(b) assessments.

Central Hudson

For permitting of the Central Hudson plant on the Hudson River, the General Counsel differentiated between the requirements of § 316(a) and § 316(b). He concluded (1977) that “Under § 316(a) the applicant has the ultimate burden of persuasion, and economic considerations are not appropriate. Under § 316(b) EPA has the ultimate burden of persuasion and economic considerations are appropriate.” Further, he concluded that § 316(a) allows for an adverse environmental impact, provided that the impact does not interfere with the protection and propagation of a balanced aquatic community. Under § 316(b) adverse environmental impact must be minimized, but only if the cost of the technology to do so would not be “wholly disproportionate to the environmental benefit to be gained.”

In Central Hudson Gas (1979) the General Counsel established that § 316(a) determinations are to be made independently of § 316(b) determinations, finding that there is no legal basis for requiring that these issues be jointly considered on one proceeding. The General Counsul opined that regulators have the discretion for each individual case even though it may be desirable to implement the respective permit conditions in a unified manner at this location.

Major implications from the Environmental Review Board (ERB)’s Brayton Point Station review

The ERB was charged with providing an expert review of EPA’s assessment of the thermal impacts of Dominion Energy’s Brayton Point Station. Although not a judicial review, the ERB’s findings are discussed here because of their relevance to any future modifications of the § 316(a) guidance.

The ERB, in its review of EPA Region 1’s contested permit for Brayton Point Station on Mount Hope Bay, Massachusetts, made several precedent-setting observations while largely affirming EPA’s analysis [Environmental Administrative Decisions 12:490-707 (2006)]. Region 1 had rejected the company’s BIP demonstration and proposed effluent limitations different from those proposed by the applicant. The applicant’s were based on an existing aquatic community in the bay that EPA judged to already be harmed by the history of thermal discharges in conjunction with effects of the water withdrawal (data and modeling indicated that the entire bay was affected by the discharge). The ERB review commented on points for Brayton Station that are applicable to § 316(a) demonstrations generally.

- **Importance of trends for evaluating harm** was stressed by both EPA and the ERB. Fisheries agencies and other groups had surveyed the abundance and composition of the bay’s fish community for several years during several phases of operation of Brayton Point Station. These surveys documented in detail an overall decline as well as specific changes in reproduction and behavior. Particularly compelling to EPA and the ERB was a marked decline in years immediately following an increase in thermal discharges (due to decommissioning of a cooling tower). [EAB p. 495, 554] Also, there were increases in abundance of nuisance ctenophores, a planktonic invertebrate that feeds on fish eggs and larvae. Further, the annual cycle of phytoplankton typical of coastal waters disappeared during station operation. with affirmative evidence of trends for or against appreciable harm when there were thermal discharges. With most U.S. waters suitable for power station cooling having been studied by independent organizations since passage of the Clean Water Act as well as periodic monitoring by the facility, such records would be part of the “best information reasonably attainable.”
- **The standard for a BIP is not the existing community, which may already be degraded.** The ERB noted that the definition of a BIP in the Clean Water Act and regulations clearly envisions a consideration of more than the population of organisms currently inhabiting the water body, citing the prior Wabash determination.
- **Importance of suitable reference areas** was clear from the ERB’s discussion of whether the baseline for a BIP at Brayton Point Station was the existing aquatic community at the time when the company submitted a variance application or a hypothetical community more typical of coastal environments (as asserted by EPA). At issue was whether the documented changes in Mount Hope Bay were regional or due, at least in part, to Brayton Point Station’s operations including thermal discharges. EPA’s assertion of what community should be there if not for the thermal discharge relied partly on the community in the main Narragansett Bay, of which Mount Hope Bay is a small part. The ERB opined that Narragansett Bay’s community, though not exactly the same as what might have been in Mount Hope Bay (it receives the outflow from the Bay and it also might be thermally affected), was an appropriate area for making comparisons of community composition. [EAB p. 555] We interpret the EAB’s discussion as supporting (if not requiring) selection of reference areas far enough removed from the thermally affected area that they reflect regional community structures and trends unrelated to the thermal discharge under consideration. EPA’s regulations (Subpart H) make allowance for irreversible changes in water bodies that would be reflected at a broader geographic scale than the thermal discharge. Reference areas are a logical part of a demonstration study and can be supplemented by similar studies by others of areas more removed from the thermal discharges.
- **Phytopankton and zooplankton can be important evidence for harm** for the aquatic community due to the thermal discharges, as shown in Mount Hope Bay. In this case, they were not Low Potential Impact biotic categories, as they are often considered. The annual spring bloom of phytoplankton disappeared during operation of Brayton Point, while predatory ctenophores became an important part of the zooplankton, which likely contributed to reductions in fish eggs and larvae. [EAB p. 554]
- **Checklists of criteria were seen by the EAB as important but not all-inclusive.** The EAB considered the regulation to be in the nature of a guideline that describes important factors to be considered at a specific location but it is not for rote application as the sole basis for decisions. [EAB p. 556]
- **In waters already harmed, the EAB opined that the demonstration must show how the conditions of the proposed variance will relieve the existing impacts.** [EAB p. 555] The EAB specifically cites the legislative history that shows the goal of the Clean Water Act is “to restore and maintain chemical, physical and biological integrity of the Nation’s waters.” [EAB p. 557]
- **Use of thermal modeling of the water body and “critical temperature exceedances” for RIS was found to be a reasonable basis for evaluating a proposed variance.** [EAB pp. 575-579]. Modeling scenarios of winter and summer conditions were used by EPA Region 1 to show significant thermal habitat degradation for Winter Flounder (*Pseudopleuronectes americanus*). An alternative variance condition stipulated that no more than 10% of the Bay should exceed 24°C in summer.
