# Supplementary material for: Modernizing thermal discharge assessments for the 21st century
Source: Integr Environ Assess Manag. 2021 Jul 22;18(2):459–68. doi: 10.1002/ieam.4472 (PMC9291154; doi:10.1002/ieam.4472)
Supplement: Supplementary file 2 — SUPPLEMENT 2. Documents case studies of four recent thermal discharge assessments that illustrate advancements in the field. [file IEAM-18-459-s002.docx]

# Supplement 2: 4 recent §316(a) studies

Brayton Point Station

Application of the 1977 guidance to the Brayton Point Station is documented in EPA Region I’s “Clean Water Act NPDES Permitting Determinations for Thermal Discharge and Cooling Water Intake from Brayton Point Station in Somerset, MA,” dated July 22, 2002. Brayton Point was a fossil-fired power plant located on Mount Hope Bay, a shallow offshoot of Narragansett Bay. Units 1, 2, and 3 were constructed prior to the enactment of the Clean Water Act of 1972. All three of these units were built with once-through cooling systems. Unit 4, built during the mid-1970s, was required to operate with a closed-cycle cooling system. Subsequently, the owners of the plant petitioned EPA to modify the discharge permit for Brayton Point to allow Unit 4 to convert to once-through cooling. A modified permit was issued in 1982, and Unit 4 began operating with once-through cooling in 1984.

As a requirement of previous permits, the facility owner began conducting trawl surveys of finfish in Mount Hope Bay in 1972. In addition, the Rhode Island Division of Fish and Wildlife (RI DFW) and the University of Rhode Island (URI) conducted regular trawl surveys of Narragansett Bay, including two stations in Mount Hope Bay. These surveys detected major declines in abundance of Winter Flounder and other fish species in Mount Hope Bay, especially following the conversion of Unit 4 to once-through cooling. Following publication of a technical report (Gibson 1996) demonstrating strong negative correlations between both heat rejection and cooling water flows and fish abundance in Mount Hope Bay, regulatory agencies began negotiating with the plant owners over permit modifications intended to reduce heat discharges into Mount Hope Bay.

As part of this process US Gen New England, Inc. (USGenNE), the facility owner, submitted an application for a § 316(a) variance. USGenNE’s proposed operating mode would retain once-through cooling of Unit 4 but would reduce the overall heat discharge from the plant to approximately the level that prevailed prior to the conversion of Unit 4 to once-through cooling. EPA reviewed USGenNE’s variance application, and subsequently performed its own independent § 316(a) Determination. Although EPA’s Determination was based primarily on empirical data and should logically be classified as a Type I demonstration, the agency structured its assessment according to the “Biotic Category Determinations” provided in Section 3 of the 1977 guidance, which deals with Type II (Predictive) demonstrations. In addition, EPA clarified the requirements of the rule, based on previous court decisions. These clarifications included:

1. “In assessing the BIP, EPA must look not only at the community as a whole but also at the effects on individual species of fish that should be part of that community.”
2. “it is not acceptable that a particular discharge will allow the propagation of *some* [emphasis original] community of fish with a certain degree of diversity and abundance; the thermal discharge limits must be sufficient to protect *the* [emphasis original] BIP that ought to be present in the particular receiving water consistent with the regulations.”

EPA also stated based on legal precedents that on a case-by-case basis the agency can focus on “discrete water bodies, water body segments, or even sub-areas within a water body segment,” and that the agency “must take into account the cumulative effects of other stresses to the BIP.”

The agency analyzed impacts on the seven Biotic Categories defined in the guidance. The assessment employed the definitions and decision criteria provided in Section 3 of the guidance and discussed in Section 2 of this report.

Phytoplankton

EPA concluded that, because seagrass and saltmarsh communities have declined, phytoplankton are now the dominant primary producers in Mount Hope Bay. Moreover, nuisance algal species have been documented in Mount Hope Bay. Therefore, the bay cannot be considered a “low potential impact area” for phytoplankton.

EPA then addressed the decision criteria listed in the guidance. With respect to nuisance algal blooms, EPA found that the occurrence of a blue-green algal bloom in Mount Hope Bay had been demonstrated using material taken from the intake screens at the Mount Hope Station. Since blue-green algae are known to be stimulated by warm temperatures, and since no comparable blooms had been documented in Narragansett Bay, EPA concluded that the thermal discharge from Mount Hope had probably contributed to the bloom, and that such blooms would probably continue in the future under the operational mode proposed by the facility owner.

With respect to phytoplankton community changes, EPA cited mesocosm research performed at URI in which effects of elevated winter water temperatures on phytoplankton, zooplankton, and mussel communities were examined. The study found that in tanks in which temperatures were maintained above the normal ambient winter temperatures in Narragansett Bay, phytoplankton abundance and biomass were lower than in control tanks. Moreover, the winter-spring phytoplankton bloom that normally occurs in the bay was observed in the control tanks but not in the tanks with elevated temperatures. In the warm tanks, settling of phytoplankton biomass into the sediment was much lower than in the cool tanks, indicating a shift in the food base from detrital production to pelagic production. EPA cited thermal analysis of satellite images and hydrothermal modeling performed by consultants to the facility owner to argue that the thermal discharge from the station was elevating winter temperatures within Mount Hope Bay sufficient to induce the same types of shifts in phytoplankton production and community structure observed in the URI mesocosm experiments. Elevated temperatures would still occur under the operational mode proposed by the owners.

Based on these analyses, EPA concluded that the operating mode proposed by USGenNE would not meet § 316(a) variance criteria for phytoplankton.

Zooplankton

EPA stated that because Mount Hope Bay serves as a spawning site for numerous fish and invertebrate species, it could not be considered a low impact area for zooplankton.

EPA’s analysis of impacts of the thermal discharge on zooplankton relied on an apparent increase in frequency of occurrence of blooms of the ctenophore *Mneimiopsis leidyi* in Narragansett Bay. The range of this species appears to be moving northward as a result of increasing coastal water temperatures. Ctenophores feed on zooplankton and compete for prey with fish larvae; studies cited by EPA have found that dramatic increases in ctenophore abundance are indicators of stressed systems. EPA argued that the incremental temperature increase in Mount Hope Bay due to the station’s thermal discharge greatly exceeds the long-term background temperature rise, therefore, the discharge is potentially facilitating the expansion of the range and seasonal bloom occurrence of *M. leidyi*. Such changes could result in increased competition for food and reduced growth and survival of larval Winter Flounder and other fish species. Based on this analysis, EPA concluded that the operating mode proposed by USGenNE would not meet § 316(a) variance criteria for zooplankton.

Habitat formers

Although eelgrass beds that were formerly present in Mount Hope Bay are now gone, efforts to improve water quality are expected to permit future restoration of these beds. Because of this potential recovery EPA concluded that Mount Hope Bay cannot be considered a low impact area for habitat formers.

Literature cited by EPA indicates that in turbid water bodies such as Mount Hope Bay, where light penetration is reduced, elevated temperatures reduce seagrass growth. EPA concluded that the Brayton Point Station discharge is contributing to the continued absence of eelgrass from the bay.

Shellfish and macroinvertebrates

EPA determined that Mount Hope Bay is not a low potential impact area for shellfish and macroinvertebrates because shellfish of commercially important species exist in the bay, because shellfish and macroinvertebrates serve as important components of this ecosystem, and because the bay serves as a spawning and nursery area for shellfish and macroinvertebrates.

EPA evaluated data from several benthic invertebrate surveys in and around Mount Hope Bay. None of these surveys detected extensive areas showing indications of environmental stress. EPA found beds of the amphipod *Ampelisca spp.* within the bay. These organisms are an important food source for juvenile Winter Flounder. Overall, EPA found no evidence of harm to shellfish and macroinvertebrate communities from either historic heat discharges or expected future discharges.

Fish

EPA determined that Mount Hope Bay is not a low potential impact area for fish because numerous species of recreational and commercially important fish species occur in the bay, because the bay is a finfish spawning and nursery area, and because there is a potential for blockage of fish migration. EPA performed its analysis of effects of the thermal discharge on the fish community in two steps. First, EPA performed a retrospective examination of total finfish abundance in relation to plant operations. Second, EPA examined specific temperature thresholds for individual species.

For the retrospective analysis, EPA evaluated long-term trends in total finfish abundance and Winter Flounder abundance in Mount Hope Bay over the period 1972-2000. Because the station was already operating in 1972, even the earliest years in the data set cannot be considered pre-operational years. However, the data set does include years before and after the conversion of Unit 4 to once-through cooling in 1984. EPA noted that “dramatic swings” in total finfish abundance occurred several times between 1972 and 1984. EPA interpreted these swings as indicating that the populations were already unstable and prone to collapse. EPA concluded that even prior to the conversion to once-through cooling Brayton Station’s thermal discharge was causing the fish community in Mount Hope Bay to become unbalanced. In addition, EPA cited an analysis performed by the RI DFW in which Winter Flounder trawl survey data from Mount Hope Bay were normalized to trawl data collected in lower Narragansett Bay and in Block Island Sound. The purpose of this analysis was to remove effects of fishing, long-term temperature trends, and other regional influences so that effects of Brayton Point’s thermal discharge could be isolated. The RI DFW analysis indicated that normalized Winter Flounder abundance in the bay declined continuously throughout the 1970s before collapsing after 1984. EPA argued based on these results that the plant operating mode proposed by USGenNE, which would reduce the thermal load from the discharge to the level that occurred prior to the conversion of Unit 4 to once-through cooling, would be insufficient to stop or reverse the decline in Mount Hope Bay fish populations.

EPA’s temperature threshold analysis compared predicted water temperatures in the thermal discharge plume with critical temperature thresholds for marine organisms obtained from scientific literature. Temperature predictions were obtained from a hydrothermal model developed by a contractor to USGenNE. After calibration to field data acquired for summer and winter conditions in the bay, the model was used to predict plume temperatures under five operational scenarios. Thermal tolerance data were compiled for 17 Representative Important Species (RIS), although most of the analysis focused on Winter Flounder.

The warmest year in the dataset, 1999, was chosen to represent ambient temperatures in the analysis. Critical temperatures for two layers (pelagic and benthic) and two seasons (summer and winter) were defined based on thermal tolerance data for the life stages of fish species expected to be present in the bay during those seasons. The pelagic layer was defined to be the upper 10 vertical layers in the hydrothermal model; the benthic layer was defined to be the bottom layer in the model. For each layer and season, a critical temperature value was selected based on the most sensitive life stage of the most sensitive species expected to be present. For the pelagic layer in summer, the critical temperature was the avoidance temperature of sub-adult and adult Striped Bass (*Morone saxatilis*), which move into Mount Hope Bay to feed during summer. For the other layers and seasons, the critical temperature values were selected based on Winter Flounder thermal tolerances. For each operational scenario, the thermal model was used to predict the volume of the bay that would exceed these critical thermal thresholds. Under the operating scenario proposed by USGenNE, substantial fractions of the bay would exceed critical temperature during all seasons except in winter. Under winter conditions, 0% of the pelagic layer would exceed the 8°C criterion for this layer, although 100% of the benthic layer would exceed the 5°C threshold established for that layer.

EPA considered three additional potential effects of Brayton Point Station’s thermal discharge. First, the presence of the warm thermal discharge in winter disrupts the normal seasonal migrations of Striped Bass and Bluefish (*Pomatomus saltatrix*). Instead of migrating south along the Atlantic coast, large numbers of these fish crowd into the discharge canal, where they are subjected to increased disease incidence and generally weakened physical condition. Second, the heated discharge may be contributing to the range extension of the Smallmouth Flounder (*Etropus microstomus*), which had recently appeared in Mount Hope Bay. Third, thermal attraction may have contributed to an impingement event in February, 2002 in which 35,000 juvenile Atlantic Menhaden (*Brevoortia tyrannus*) were impinged on the intake screens of Units 1, 2, and 3.

Other vertebrate wildlife

After consulting with the National Marine Fisheries Service and with a scientist from URI, EPA determined that Mount Hope Bay is not a significant habitat for marine mammals or sea turtles. Based in this finding, EPA concluded that Mount Hope Bay is a low potential impact area for vertebrate wildlife.

Cumulative impact assessment

Section 316(a) requires EPA to consider, in addition to thermal impacts, impacts of other stressors as well. To meet this requirement EPA performed a cumulative impact assessment that considered interactions of the Brayton Point thermal discharge with five other stressors that were potentially affecting Mount Hope Bay: overfishing, predators, water quality, brown tides, and intake structure impacts (i.e., entrainment and impingement). EPA concluded that the thermal discharge had exacerbated the effects of all of these stressors except brown tides.

Final determination

Based on its analysis EPA determined that the operation of Brayton Point Station had not maintained a Balanced Indigenous Community within the bay, and that future operations under the owner’s proposed scheme would not lead to restoration of the BIC. The primary reasons cited by EPA were (1) the dramatic loss of total finfish abundance and biomass, with 16 of the 21 species in Mount Hope Bay having declined greatly, and (2) increased prevalence of thermotolerant species such as ctenophores and Smallmouth Flounder.

EPA denied the owner’s request for a variance under § 316(a) and proposed a set of alternative discharge limits that the agency claimed were less stringent than either technology-based or water-quality based limits but would still be protective of the BIC.

EPA’s determination was not the final statement, however. The decision was appealed to EPA’s Environmental Appeals Board, which rendered a detailed report on the case (EAB 2006). The EAB generally accepted Region 1’s analysis and conclusions while commenting on issues of broad application to § 316(a) demonstrations.

Implications for future guidance

The Brayton Point Station case has had major implications for no-prior-harm studies and demonstrations, which have been incorporated into the concepts for modernizing guidance provided in this report. For example, there was a significant conceptual shift from the demonstration of a static BIP/BIC in the thermally affected zone that “will allow the propagation of *some* [emphasis original] community with a certain degree of diversity and abundance” to “thermal discharge limits [that are] sufficient to protect *the* [emphasis original] BIP that ought to be present in the particular receiving water consistent with the regulations.” Therefore, “balance” is a relative measure. This supports the concept of applying increased emphasis on having adequate reference areas for quantitative evaluation of what “ought to be present” including third-party studies.

In addition, the EPA re-analysis had a strong emphasis on demonstrating impacts on biotic categories (as defined in the 1977 guidance) in Mount Hope Bay compared to other parts of Narragansett Bay. This involved detailed evaluation of whether each category could be considered as having Low Potential Impact based on actual data comparisons. The traditional guidance of likely Low Potential Impact for phytoplankton, for example, was contradicted for this site. For phytoplankton and zooplankton, both mechanistic updates (thermal damage during high-temperature exposures in thermal plumes) and consideration of the roles of phytoplankton and zooplankton in critical ecosystem processes may be considered.

The EPA viewed cumulative ecosystem effects as total ecosystem responses (e.g., shifts in community composition and ecosystem processes from that seen in reference areas) as well as the usual evaluation of interactions of added heat with other stressors (e.g., dissolved oxygen, toxicants, overfishing, predators).

The Brayton Point Station analysis relied on both retrospective and predictive elements. Despite a strong, comparative whole-ecosystem emphasis, the analysis also focused on key species, such as relative decline in 21 fish species and increase in thermally tolerant ctenophores in the zooplankton. Their “temperature threshold analysis” of RIS from the literature compared thermal tolerance to temperatures predicted by hydrothermal modeling. This supports the concept of pursuing multiple lines of evidence in demonstrations, including both retrospective and predictive components.

The checklist of decision criteria was useful but had to be relevant locally. For example, disruption of seasonal fish migrations along the eastern seaboard was highlighted as a significant impact, acting as a reminder that all sites have somewhat unique vulnerabilities. While we recommend that guidance provide a basic checklist, there should be thoughtful consideration of other criteria that may be locally significant.

Cardinal Plant

In January 2017, a § 316(a) demonstration for the Cardinal Plant in Jefferson County, OH was submitted to the Ohio Environmental Protection Agency (OEPA) by American Electric Power (AEP). Cardinal is a fossil-fired power plant located in Jefferson County, Ohio on the Pike Island Navigational Pool of the Ohio River. Units 1 and 2, both of which began commercial operation in 1967, utilize once-through cooling. Unit 3, which began operation in 1977, utilizes closed-cycle cooling.

The 2017 Demonstration is an update of a previous demonstration that was approved by OEPA in 1977. The earlier demonstration was a predictive (Type II) demonstration because no biological sampling results from the vicinity of the Cardinal Plant were available. Information on fish species likely to be present near the plant was obtained from lock chamber rotenone samples collected by and the Ohio River Valley Water Sanitation Commission (ORSANCO) above and below the Pike Island Pool. Temperature tolerances of fish species identified from the ORSANCO samples were evaluated from published literature and compared to measured or estimated thermal plume temperatures. The 2017 update was a retrospective (Type I) demonstration that relied on field studies conducted by AEP, the Ohio River Ecological Research Program (ORERP), and ORSANCO from 1978 through 2016.

The 2017 Demonstration was performed under the Ohio EPA (OEPA)’s 1978 § 316 guidance. Similar to the definition provided in the federal § 316(a) guidance, the OEPA defines the BIC as:

“…an assemblage of balanced species populations living in a prescribed physical habitat that have a definite functional unity, characteristic trophic structure, patterns of energy flow, and a compositional unity in that there is a certain probability that certain species will occur together.” (OEPA 1978, p. 7)

OEPA’s guidance is not organized around biotic categories like those defined in Section 3 of the federal guidance. Instead, OEPA’s guidance defines 5 basic attributes of a BIC:

- All trophic levels necessary to the functioning of the community are present (includes necessary food web organisms);
- Non-domination by pollution (including thermal) tolerant organisms, unless their presence is expected or cannot be directly attributed to environmental stress;
- Expected species are present and are in numerical proportion (relative abundance) to each other indicating the environmental stress is absent or minimal;
- The ability of representative species to reproduce in numbers sufficient to maintain levels of relative abundance, and;
- Previous trends (taking into account normal dynamic patterns) in community diversity, abundance, composition, and well-being are maintained.

The 2017 Demonstration (pp. 52-53) notes that OEPA’s concept of a BIC assumes that biological interactions among and between faunal groups tend to return community structure to a state of balance following disturbance; “unbalance” occurs when the ability of self-regulating mechanisms compensate for an environmental stress is lost. The Demonstration argues that although this concept of balance may apply to some ecosystems, many systems are disturbance-dominated and do not possess definable balanced states. The Demonstration cites published literature as showing that riverine systems are among those that are disturbance-dominated. These disturbances must be taken into account when applying OEPA’s attributes of balance to the thermal discharge from the Cardinal Plant. Having expressed this caveat, AEP’s demonstration attempted, using empirical data collected over many years of monitoring, to demonstrate that all five of OEPA’s attributes of a BIC are present in the vicinity of the Cardinal Plant.

Assessment Approach

The Pike Island Navigational Pool, excluding a thermal plume mixing zone approved by OEPA, was identified as the location where the BIC must be protected. Biological, water quality data were available from stations both above and below the plant.

- Seasonal sampling of adult and juvenile fish was conducted by electrofishing during all years between 1978 and 2016 except for 2002. In some years, nets, trawls, and seines were used in addition to electrofishing.
- Benthic macroinvertebrate sampling was collected during 12 years using Hester-Dendy artificial substrate samplers and Ponar grab samplers.
- Selected water quality variables were measured at all fish collection locations.

Qualitative mussel surveys were conducted during four years, and ichthyoplankton tows were conducted from 1978-1990, however, data from these surveys were not used in the assessment.

Three types of quantitative analyses were performed as part of the assessment: detailed analysis of data collected during the low-flow years of 1988 and 2012; upstream vs. downstream comparisons of fish community metrics; and upstream vs. downstream abundance comparisons for representative aquatic species (RAS).

Discussion of significant low-flow years

Ohio River flow rates at the Pike Island Lock and Dam during the summers of 1988 and 2012 were substantially lower than the long-term averages. Water temperatures during those years were correspondingly elevated.

In June through August 1988, temperatures measured below the station were 3-8°C higher than above the station, however there was no difference in dissolved oxygen concentrations between these two zones. Macroinvertebrate sampling was conducted in June and August both upstream and downstream from the plant, using two sampling gears. There were no consistent differences between upstream and downstream locations with respect to total taxa, mean density, or Shannon-Wiener diversity. Larval fish beach seining was conducted at two sites upstream and one site downstream from the discharge. Larval fish density was higher at the downstream site than at either of the upstream sites. Numbers of fish species collected during the adult/juvenile fish surveys were essentially the same upstream and downstream from the station, and several of the species collected downstream are considered to be thermally sensitive.

In 2012, measured water temperatures again were higher downstream than upstream from the discharge. Dissolved oxygen concentrations were consistently lower downstream than upstream of the discharge, but still were well above ORSANCO’s aquatic life criteria. No benthic macroinvertebrate or larval fish data were collected in 2012. However, analysis of the adult and juvenile fish surveys showed that (1) only two of nine fish community metrics displayed significant spatial (upstream vs. downstream) differences, and (2) there were no consistent spatial patterns in catch rates for eight thermally sensitive fish species, in spite of the elevated downstream temperatures. Independent fish monitoring data collected by ORSANCO in 2012 showed that aquatic life use criteria for the Pike Island Pool were being attained, in spite of the drought conditions. ORSANCO also noted that there had been significant habitat changes within the pool due to dense growths of submerged aquatic vegetation. These habitat changes may have caused a shift in fish community structure compared to earlier years.

Statistical analysis of long-term fish collection data

Spatial comparisons (upstream vs. downstream) of community metrics and catch data for individual species were performed using the adult/juvenile fish survey data. Only data collected in 1991 and later were used, because 1991 is the year electrofishing surveys switched from daytime sampling to nighttime sampling. A species richness analysis using data from 2005-2014 showed that there were seasonal differences in species richness with fewer species being collected in June than in August or October. However, there were no differences between upstream and downstream richness during any of these months. Averaged over the years 2005-2014, values of the “modified Index of Well-Being” (IWBmod) used by OEPA to evaluate attainment of aquatic life uses were somewhat lower downstream of the Cardinal Plant than upstream in August, but not in June or October. However, median IWBmod values for both upstream and downstream locations achieved the minimum biological criteria during all months.

Values of the Ohio River Fish Index (ORFIn) used by ORSANCO to assess attainment of aquatic life use in the various navigation pools were also calculated. Over the period 2005-2014, ORFIn values for downstream locations were slightly lower than for upstream locations, but the scores for all locations exceeded the threshold for attaining aquatic life use.

With respect to long-term trends in community metrics, over the period 1991-2014 species richness values and ORFIn values averaged over all stations increased; there were no trends in IWBmod scores.

With respect to catch rates of 12 common species over the period 1991-2012, three exhibited higher catch rates upstream from the Cardinal Plant, four species exhibited higher catch rates below the Cardinal Plant, and five species exhibited no differences between upstream and downstream catch rates.

Evaluation of Representative Aquatic Species

As a supplement to the community analyses described above, AEP evaluated thermal effects on 12 fish taxa identified as “representative aquatic species” (RAS). A total of 11 of these taxa were individual species; the 12^th^ was the genus *Morone*, including White Bass (*Morone chrysops*) and hybrid Striped Bass (*M. saxatilis x M. chrysops*). These RAS included fish inhabiting both benthic and pelagic habitats, and representing multiple trophic guilds, including planktivores, insectivores, omnivores, and piscivores. Data used in this analysis was for the years 2005-2014. One species, Mooneye (*Hiodon tergisus*), was to uncommon for inclusion in the analysis. Significantly lower downstream catch rates were observed only for Golden Redhorse (*Moxostoma erythrurum*), *Morone sp.*, Sauger (*Sander canadensis*), and Freshwater Drum (*Aplodinotus grunniens*). Measured temperatures downstream from the plant were well below thermal mortality thresholds for these species, but temporary avoidance of the warmest locations in the Pike Island Pool may have occurred on some occasions.

Application to the OEPA § 316(a) Decision Criteria

AEP addressed the OEPA decision criteria based on the above monitoring results. Based on the absence of observable adverse effects that could be related to plant operations, AEP argued that all five of the attributes of a balanced community were being met in the vicinity of the Cardinal Plant:

“All trophic levels necessary to the functioning of the community are present (includes presence of necessary food web organisms).

The fish and invertebrate communities near Cardinal Plant contain all trophic levels necessary for normal, expected ecological persistence and function.

Non-domination of the community by pollution tolerant species, unless their domination is expected and cannot be directly attributed to thermal stress.

Fish community scores upstream and downstream of Cardinal Plant attain Ohio River aquatic life use criteria and there is no evidence that communities downstream of the once-through cooling discharge are dominated by those species considered tolerant.

Expected species are present and in numerical proportions to each other that indicates that adverse thermal stress is minimal or absent.

IWBmod scores are similar at upstream and downstream locations. During the hottest sampling period (August), there appears to be evidence of temporary avoidance for some thermally-sensitive species. This avoidance, however the magnitude, does not translate into lowered fish community index scores at downstream locations. If and when avoidance by thermally-sensitive species does occur, the size of the Ohio River is such that these fish can take refuge in cooler segments without adverse stress.

The ability of representative species to grow, survive, and reproduce in numbers sufficient to maintain levels of relative abundance

With the exception of one RAS (Mooneye) that is naturally rare in the upper Ohio River, all other RAS are persistent and present in expected numbers near the Cardinal Plant.

Previous normal trends (taking into account normal dynamic patterns) in community structure, diversity, well-being, and composition are maintained.

The segment of the Ohio River downstream of the Cardinal Plant once-through cooling discharge displays the same temporal trends in improved water quality and more diverse biological communities as segments upstream of the plant, or at navigation pools upstream and downstream of the Pike Island Navigation Pool. With the exception of 2002, water quality and ecological studies have been conducted near Cardinal Plant since 1978. This sampling frequency would certainly have detected appreciable, unacceptable adverse effects of these could be solely attributed to the once-through cooling discharge.” (AEP 2017, pp 94-96)

The OEPA accepted AEP’s variance request and the Cardinal Plant continues to operate according to the operating mode proposed by AEP.

Implications

The Cardinal plant is an example of a § 316(a) analysis and demonstration that followed state (Ohio) guidelines and decision criteria rather than the federal ones. Implementation of § 316(a) has been delegated to the state. Nonetheless, the demonstrations have been found acceptable to EPA. This assessment and several notable attributes.

First, the latest demonstration relied on data from multiple sources, including the utility (AEP), the coordinated studies by Ohio river utilities under sponsorship of EPRI’s ORERP, and the long-standing river basin commission ORSANCO. Such regional coordination is laudable.

Second, the latest demonstration made use of a series of data sets from 1978 to 2016. Thus, the demonstration examined trends in community structure, which is especially helpful and a characteristic we recommend.

Third, guidelines from the state of Ohio’s EPA were the basis of the demonstration. The Ohio guidelines reinterpret the basic evaluation structure and criteria of the federal regulations and 1977 guidance. The specific federal criteria are reworked into the four listed above. Evaluation was not focused biotic categories as advised in the 1977 EPA guidance. In contrast to strict adherence to the federal criteria expected by EPA Region 4 (Southeast), EPA Region 5 seems to be more lenient. For example, efforts by the Tennessee Valley Authority to use its multi-factorial indices of aquatic health (developed in cooperation with regional state agencies) were not accepted by EPA Region 4 for the Cumberland Fossil Plant, Tennessee. Such regulatory reworking isn’t the norm nationally, and companies should consider obtaining clarity on the basic structure and criteria from the appropriate agency before beginning any study and demonstration preparation.

Labadie Energy Center

Several unusual or unique features of a § 316(a) demonstration are represented in the permitting of Ameren Missouri’s (Ameren) Labadie Energy Center (LEC) (Ameren 2019). The LEC is an early 1970s steam electric power plant located in Labadie, Missouri on the south bank of the lower Missouri River (LMOR) near River Mile (RM) 57 in Franklin County, 35 miles west of St. Louis, Missouri. The LEC has four generating units, each with two circulating water pumps, and a gross generating capacity of 2,580 megawatts (MW). The LEC utilizes once-through cooling and withdraws water for each unit from the Missouri River via a shoreline intake structure. The resulting heated effluent is discharged to a 1,400-foot-long artificial discharge canal and the adjacent navigation channel of the lower Missouri River (LMOR). This discharge of heat is regulated through a National Pollutant Discharge Elimination System (NPDES) permit (MO-0004812) issued by the Missouri Department of Natural Rsources (MDNR).

Biological studies and hydrothermal modeling were first performed in the mid-1970s as part of the facility's initial NPDES permit application. Those studies concluded that the LEC was a site of low potential impact for all biotic categories. An NPDES permit was issued in 1977 which included an approved alternative effluent limitation (variance). Biological studies were voluntairly and periodically performed over the years. MDNR renewed the § 316(a) variance over several permitting cycles until 2015 when MDNR declined to re-issue the variance pending additional biological studies.

An overall NPDES permit was issued to the LEC with an effective date of August 2015 that included an interim thermal effluent limitation that was to remain in effect through July 31, 2025. On August 1, 2025 the facility would have been required to meet final effluent limiations equivalent to the temperature criteria in the Missouri water quality standards (MWQS_t_). In addition, this permit required the LEC to reestablish a biological monitoring program to evaluate the potential impacts of the thermal discharge on aquatic communities. A full biothermal study had not been conducted since the 1970s, and critics of the permit were calling for one. In 2017, MDNR approved a biological study plan as part of Ameren's efforts to renew its NPDES permit application and to support a new variance request. In order to accommodate the overall permitting schedule, a preliminary analysis of monthly collections of biological field data from 2017 was conducted (comparison of species richness, species identities, and numbers of each species in two reference zones and the thermally affected zone for for fish, ichthyoplankton and macroinvertebrates). It also included comparison of the 2017 results with previous studies at the site. As a result, the interim thermal effluent limitations in the 2015 permit were terminated, and the final thermal effluent limitations of the permit became effective on August 1, 2018.

In keeping with current EPA expectations (not formalized in early EPA guidance), the Alternative Effluent Limitation was proposed by Ameren and included in the demonstration. This limitation is rather unique in that it incorporates river flow, discharge flow, and effluent and river river temperatures in a daily index (Thermal Discharge Parameter, TDP; see Ameren 2019 for derivation of the formula). The rationale is that the liklihood of the thermal discharge causing harm to the river biota depends on its volume and temperature relative to the source water at the time. Both the EPA expectations and the logic suggest to us that this is an important precedent. The Alternative Effluent Limitations is:

- A TDP of greater than 0.95 will be allowed under conditions when the river flow is less than 40,000 cubic feet per second (cfs) or ambient river temperatures are greater than 87°F;
- A TDP of greater than 0.95 will be allowed in no more than 5 percent of the days in any calendar year; and
- On any day where the TDP is greater than 0.95, the mixing zone must be less than 40 percent of the volume of the river as calculated by the equations in the permit.

The Labadie case is unusual, also, in having two independent consultants evaluating the field data and its interpretation in relation to the lower Missouri River biota. The Sierra Club appealed the MDNR’s 2015 interim permit for not complying with the federal Clean Water Act and Missouri Clean Water law. Although the thermal discharge met the Missouri water temperature standards most of the time in the past several years, there were occasional exceedences above TDP of 0.95 when flows were low and ambient river temperatures high. The Sierra Club asserted a general harm from raising river temperatures, citing generic literature on thermal effects and some published papers that they claimed (often incorrectly) that river temperature was the dominant factor determining the life cycles of Missouri River aquatic life (river flow and channelization are actually recognized as main determinants, with seasonal temperature also important). They also did not consider the hydraulics of effluent mixing in the river. The timing of the legal proceeding for the Sierra Club appeal necessitated an initial analysis of the first year of field data before the two-year study and the demonstration were completed. A second consultant was enlisted for the early analyses and presentation of expert testimony to the administrative hearing. This allowed simultaneous uninterrupted field collections and analyses by the primary consulting firm. Ameren thus continued its contracted two-year field study and used it for its final demonstration supporting a variance on the basis of no prior appreciable harm even with the occasional exceedences.

The approved study plan was a Type I field study with engineering, RIS, and other relevant information, per the 1977 guidance. It is informative for including two full years of field collections, two reference locations (upstream and downstream beyond the physical thermal plume), sampling zones selected based on initial thermal modeling, detailed sampling locations selected to match river morphology (channel, inside bends, outside bends, sand bars, relationship to river control structures), and multiple sampling technologies for each biotic category that was sampled (macroinvertebrates, fish). Jusitification for Low Potential Impact designation of phytoplankton and zooplankton (other than ichthyoplankton) was prepared during preparation of the study plan and its review and acceptance by the MDNR. The Low Potential Impact was justified for the river environment using criteria provided in the 1977 guidance (phytoplankton that was not the food base for the riverine ecosystem, low numbers of zooplankton, lack of meroplankton) and rapid passage through the plume. Those justifications became part of the final demonstration.

The demonstration was truly retrospective in that it incorporated the history of repeated field studies at the site. Studies in the early 1970s included all biotic categories, with one upstream site as a reference area. There was a sequence of fish electrofishing in upstream and thermally affected zones that allowed an analysis of temporal trends in several fish populations.

Both the demonstration and the opposition to the Sierra Club’s permit appeal made use of several broad-scale studies published by others (federal, state, and academic teams) of the ecological health of the channelized and diked Lower Missouri River in which the LEC’s thermal effluent is discharged (Angradi et al. 2009, 2011; Berry et al 2004; Galat et al. 2005). These studies provided a thorough inventory of the fish species found in the Lower Missouri River as well as multi-year trends in their abundance, and independent data for comparison of the macroinvertebrates and fish in reaches upstream and downstream of the LEC. These studies provided a line of evidence for *reference conditions* in the Lower Missouri River to supplement evidence from *reference sites or zones* in the Labadie study. These third-party studies were analogous to the several agency studies of Narragansett Bay and Mount Hope Bay that provided third-party, evidence for the reference conditions that were crucial for the Brayton Point decision (EAB 2006). Similarly, a history of federal, state, and academic studies on the Ohio River contributed to the case for no appreciable harm at the Cardinal Plant.

Having two reference zones, upstream of the thermal plume and downstream beyond the measurable plume, helped quantify any anomalous patterns in the thermally affected zone. Features of the thermally affected community (species richness, species numbers, taxonomic identities, diversity, etc.) were compared to the range of those features between the two reference zones. Differences between the reference zones could be attributed to natural or sampling variability and thus roughly quantify the relevance of differences between thermal and either reference zone.

A check-list of evaluation criteria in the 1977 guidance and further developed over many years of evaluating thermal discharges (Coutant 2019; sections 2 and 5.2 of this report) was helpful for clarifying the study results. The list with conclusions for each is presented here, as it appeared in testimony for the hearing appeal [edited slightly to reflect the full demonstration].

- *Inclusion of all trophic levels-* Fish guilds representing the range from benthic detritus feeders and primary consumers to top predators are present in the thermally affected zone similar to the reference areas and those to be expected in the Lower Missouri River.
- *Diversity-* There is high species richness in the thermally affected area similar to the reference areas.
- *Capability to sustain itself-* There is evidence from the broad studies that most species’ populations found in the Lower Missouri River are “Secure” (Galat et al. 2005) meaning that they are successfully reproducing year after year. There were large increases in numbers captured in late summer and fall in both reference areas and the thermally affected zone in the LEC’s 2017 study, particularly juveniles and schooling species, indicating successful reproduction in the thermally affected zone.
- *Presence of necessary food-chain species*- Relatively small, schooling species (e.g., chubs, shiners, Gizzard Shad [*Dorosoma cepedianum*], Goldeye [*Hiodon alosoides*]), which are the main “forage” species, are abundant near the LEC in both the reference and thermally affected zones
- *Lack of domination by pollutant-tolerant species-* The species in the vicinity of LEC, including reference areas and the thermally affected area, reflect essentially the same “big river” fish assemblage.
- *Indigenous species increase or decrease-* Concurrent with invasion of the Lower Missouri River by exotic Asian carp species, there has been some decline in cool-water fish species in the Lower Missouri River, but not particularly in the vicinity of LEC. It is generally believed by regional biologists that the decline is due largely to the invasion of the exotic species, which is unrelated to LEC’s thermal discharges.
- *Threatened & Endangered Species status, with increase or decrease-* The Pallid Sturgeon (*Scaphirhynchus albus*) is the only federally endangered species potentially in the vicinity of the LEC, and its status is declining throughout the Missouri River unrelated to LEC’s thermal discharge. The Shovelnose Sturgeon (*S. platorynchus*), is listed as threatened, not for its own sake but as a protection of the nearly identical Pallid Sturgeon. The two species are difficult to separate without detailed morphological information only obtainable on a laboratory specimen (not feasible for field identifications). The Shovelnose Sturgeon seems to be a regular inhabitant of the LEC vicinity. The Lake Sturgeon (*Acipenser fulvescens*) is a state-listed species, generally found far upstream of the LEC, which has not been found in the 2017 studies.
- *Biological data on key species (T&E, prominent)-* Presence of fish species in the vicinity of LEC appears to follow physical habitat preferences rather than any responses to particular thermal regimes. The final study report for the 2-year study [included] literature data on key species’ environmental requirements.
- *Presence of Critical Function Zones in thermally affected area-* The thermally affected zone (thermal plume) does not contain any critical function zones not also represented abundantly in the rest of the nearby river.
- *Habitat exclusion by temperature-* The species occurrence and numerical data for 2017 [and 2018] show that the thermally affected area of the river does not exclude fish occupancy by elevated temperatures (occurrences in the months when temperatures are highest are similar to reference areas).
- *Thermal effects on “unique or rare habitat”-* The habitats in the thermally affected zone are not “unique or rare” for this portion of the Lower Missouri River, and do not demonstrate significant thermal effects on species occurrence or numbers collected.
- *Habitat former alterations-* The Lower Missouri River in the vicinity of LEC, both reference and thermally affected areas, does not have biotic components needed by fish and invertebrates as habitat (e.g., aquatic vegetation beds) that are altered by elevated temperatures (study team). Angradi et al. (2009) notes that the entire lower river is essentially devoid of submerged aquatic vegetation.
- *Trends in the aquatic community*- The 1974-1975 monitoring study at Labadie provides an opportunity to see if there have been changes in aquatic populations over time**.** There have been species changes, the most pronounced being the invasion of Asian carp species and related decline in some native species, all of which are unrelated to the LEC’s thermal discharges. Decline of the thermally tolerant and nuisance common carp downstream of the LEC suggest improved river conditions. The [two-year] results of the current Study Plan are consistent with the 1974-75 analysis performed at LEC, discussed above, and reflect the preponderance of typical “big river” fish species assemblages in the channelized and diked Lower Missouri River. These include Gizzard Shad, Gar, Common Carp (*Cyprinus carpio*), River Carpsucker (*Carpiodes carpio*), buffalo species (*Ictiobus* spp.), Channel Catfish (*Ictalurus punctatus*), Flathead Catfish (*Pylodictis olivaris*), White Bass, and Freshwater Drum (as described by the MDNR).
- *Nuisance species abundance*- The Lower Missouri River is being colonized by exotic Asian carp species (Silver Carp [*Hypophthalmichthys molitrix*], Bighead Carp [*H. nobilis*], Grass Carp [*Ctenopharyngodon idella*]) through a natural process of range expansion from the Mississippi River basin not caused by thermal additions at LEC. They are occurring and spreading farther upstream than LEC. The other principal nuisance species, the Common Carp, is actually on a slight decline, consistent with reduced stressors in the lower Missouri River, also unrelated to LEC’s thermal additions.
- *Zone of passage around the thermal plume* - The thermal plume occupies less than 25% of the cross section of the river, thus providing ample zone of passage for fish movement upstream and downstream should the thermally affected area be avoided. Data collected in the current study indicates that the thermal plume area is not avoided.
- *Change in commercial or sport fisheries*- The species identified by others as of commercial or sportfishing importance in the Lower Missouri River are present and reasonably abundant.
- *Magnitude and Duration of any identifiable thermal effects*- The data currently available indicate that any thermal effects on fish species richness and abundance are of low magnitude or short duration and not persistent in either space or time.
- *Sub-lethal or indirect effects*- Temperature increases above ambient certainly can cause some small, sublethal (often favorable) physiological effects and behaviors different from those in ambient waters, based on centuries of physiological and behavioral research (Precht et al. 1973; Wieser 1973). Most important, as EPA recognizes, is the magnitude and duration of such effects. At most temperatures seen in the thermally affected zone at the LEC outside of the artificial discharge canal, the temperatures are within the normal thermal range of the big-river species, and the durations of exposure to the warmest temperatures are too short to cause significant change in physiology. The presence and abundance of aquatic life in the study area suggest that these effects are not detrimental to the fish and maroinvertebrate species assemblages and reproductive successes.
- *Interaction of the thermal discharge with other pollutants*- The presence and abundance data do not indicate any detrimental interactions.

Thus, the demonstration provided evidence that all the usual relevant criteria for a BIP/BIC included in regulations, the 1977 guidance, and further elaboration based on administrative history are being met and that a BIP exists in the thermally affected zone (no prior appreciable harm), notwithstanding LEC’s thermal discharges.

Evergreen Packaging’s Canton Mill

Not all thermal effluents are discharged from electricity generating stations. The EPA regulations at 40 CFR 125.70 state that their purview of thermal discharges covers “any point source otherwise subject to the provisions of section 301 or section 306 of this [Clean Water] Act.” Thus, heated effluents from industries such as paper mills also qualify. Evergreen Packaging’s Canton Mill provides an example of some unique considerations for obtaining an Alternative Thermal Effluent Limitation (variance) and exemplifies features of the required demonstration that may be applicable to any thermal discharge to a small river.

Evergreen’s Canton Mill is a paper mill located at River Mile 63.4 on the Pigeon River at Canton, North Carolina. The Pigeon River is a small river that is a tributary of the French Broad River, which is a major tributary forming the Tennessee River. The Mill draws water for papermaking processes from a low-head impoundment on the Mill site. It is permitted by NC Department of Environment and Natural Resources to discharge 29.9 million gallons per day (MGD; 46.3 cfs) of treated industrial waste, treated domestic waste, storm water and landfill leachate to the river, which averages 325 cfs flow annually at the Mill. The 7Q10 at the Mill is 52 cfs in summer and 63 cfs in winter. The river downstream of the Mill has a history of extreme chemical pollution in addition to added heat since the early 1900s. The river in the approximately eight miles from the outfall to the headwaters of Waterville Reservoir, a hydropower reservoir, was nearly devoid of aquatic life until the early 1980s when major process improvements removed nearly all chemical pollutants, increased dissolved oxygen in the outfall and river, and reduced the heat load.

Two riverine zones were of concern for demonstrating a BIP/BIC in a 2012-2013 § 316(a) biothermal field study in support of a “no prior appreciable harm” variance demonstration: a thermal mixing zone in about the first half mile downstream of the outfall, and the zone of heat dissipation from there to the reservoir where the gradually diminishing thermal influence of the Mill is lost. The 2012-13 study included temperature measurements and modeling in the plume and river from upstream of the Mill to Waterville Reservoir, and biological studies at eight stations from the Mill to the reservoir plus reference stations.

The demonstration (Wilson et al. 2014) was organized so that attention to the specific requirements of the regulations (diversity, capacity to sustain itself, necessary food-chain species, lack of domination by pollution-tolerant species) and other relevant criteria (e.g., zone of passage, habitat formers) was clearly evident in the Table of Contents and easily identifiable text. Experience with EPA and NCDEQ reviews of earlier demonstrations indicated that such specific coverage can be difficult for a reviewer to find when not clearly identified in a long document.

There are several notable features of the 2012-13 study:

1. The study was the fifth in a series of similar studies (1987, 1995, 2000, 2005). This series provided an opportunity to examine trends in species occurrence and abundance over the time period when alternative thermal effluent limitations were included in the Mill’s permit.
2. The study, as well as prior ones, focused on the biological condition of the riverine community in the late summer and early fall of one year at the end of the major reproduction season (evidence of population sustainability). Benthic macroinvertebrates and fish were the main quantitative focus, with observations on periphyton in the plume, rooted aquatic plants, and wildlife (including crayfish and salamanders).
3. Thermal conditions were defined by field temperature measurements in the plume and in the river from upstream of the Mill to Waterville Reservoir in summer and winter. The measurements were used to calibrate a CORMIX thermal plume model and a one-dimensional model of temperature elevation above ambient (delta T) to Waterville Reservoir in summer and winter. Calibrated models gave the capacity to estimate temperature rises under conditions not actually measured (e.g., 7Q10 flows). Modeled and measured temperatures were correlated with the biological communities at the several monitoring stations and predictions of effects under temperature extremes. There were community effects in the thermal plume that correlated well with river temperatures and known thermal relationships (e.g., summer observations of fish avoiding warmest areas and using the cooler zone of passage, and benthic growths of blue-green algae in the warm plume). The low Mill-related delta T values in much of the river downstream of the plume correlated well with lack of differences with reference sites.
4. The study included multiple reference stations to compare with the biota in the river downstream of the Mill. There were six reference stations in the Pigeon River and major tributaries upstream of the Mill and two in the nearby Swannanoa River within the French Broad River watershed. Several quantitative measures were used to identify differences between the heat-affected river locations and multiple reference sites.
5. The study included reintroductions of indigenous species that had been extirpated during the early history of the Mill and whose recolonization after water quality improvement was impeded by upstream and downstream dams (e.g., darters, mussels). This interagency cooperative effort was supported by Evergreen and regional federal and state resource agencies. The reintroductions tested the premise that several indigenous species found in nearby rivers could survive and thrive in the Pigeon River under the permitted alternative effluent limitations. Since the discharges of many paper mills have a history of degrading the receiving water, such reintroductions may be a valuable mitigation toward restoring a BIP/BIC elsewhere.
6. Thermal (and general pollution) sensitivities of individual species of interest (RIS, T&E species) were reviewed and related to the current (2012) occurrence and abundance, and trends over the 1987-2012 series of studies. In particular, two measures were indicative of river improvement: (1) the changing ratio of abundance of Redbreast Sunfish (a pollution and thermally tolerant, non-indigenous species that showed relative decline) to its ecological competitor Rock Bass (*Ambloplites rupestris*; native, pollution and thermally sensitive species that showed relative increase), and (2) the declining relative abundance over time of Common Carp (nuisance, pollution and thermally tolerant, invasive) in the fish community relative to more sensitive Smallmouth Bass (*Micropterus dolomieu*), the major sports fish. Although the regulations refer to “domination by pollution tolerant species” [40 CFR 125.71(c)], EPA reviews have consistently focused on thermal tolerance. The improving biotic community could be related to general water quality improvement as well as absence of harm due to the added heat.
7. Some interactions of the added heat with other pollutants typical of paper mills were noted in each year’s study, especially oxidation of residual dissolved organic matter in the discharge leading to potential decreases in riverine dissolved oxygen. As a result, Evergreen adds oxygen to the effluent and to the river about two miles downstream yielding river concentrations that meet state standards of 5 mg/L.

Multiple measures provided evidence of continual improvement in the biological condition of the river downstream of the Mill over 25 years of alternative effluent limitations and periodic biothermal studies. In 2012, there were insignificant differences between heated and reference communities beyond the thermal plume. Several once-extirpated species that have been reintroduced (including the one identified endangered species in the watershed) have survived, grown, and (for fish and snails) reproduced. Interpretation of a BIP/BIC in the context of local environmental recovery has yielded a river now prized for a smallmouth bass sports fishery and heavy use for recreational rafting.

Implications

Several of the topics covered in our paper were informed, in part, by experience with the Canton Mill’s past two demonstrations. These include the value of checklists of assessment criteria to guide studies and demonstration documents (and help reviewers), the importance of multiple reference sites and third-party studies for determining the composition and variability of unheated communities for comparison with the thermal discharge, the value of thermal modeling for estimating extreme thermal conditions for use with thermal tolerances of RIS, and the importance of trends especially for progressive improvement in thermal criteria. More generally, we believe that the evidence presented for the Canton Mill demonstrates the value of our 21^st^ Century redefinition of a BIP/BIC, which clarifies the notion of “balance” that is in the CWA and the EPA regulations.

References

1. Ameren Missouri. 2019. Labadie Energy Center. § 316(a) final demonstration. St. Louis, Missouri.
2. American Electric Power (AEP). 2017. Cardinal Operating Company – Cardinal Plant updated § 316(a) alternative effluent limitation demonstration.
3. Angradi, T. R., D. W. Bolgrien, T. M. Jicha, M. S. Pearson, B. H. Hill, D. L.Taylor, E. W. Schweiger, L. Shephard, A. R. Baterman, M. F. Moffett, C. M Elonen, and L. E. Anderson. 2009. A bioassessment approach for mid-continent great rivers: the Upper Mississippi, Missouri, and Ohio (USA). Environmental Monitoring and Assessment 152:425-442. DOI 10.1007/s10661-008-0327-1
4. Angradi, T. R., D. W. Bolgrien, T. M. Jicha, M. S. Pearson, D. L. Taylor, M. F. Moffett, K.A. Blocksom, D. M. Walters, C. M. Elonen, L. E. Anderson, J. M. Laxorchak, E. D. Reavie, A. R. Kireta, and B. H. Hill. 2011. An assessment of stressor extent and biological condition in the North American mid-continent great rivers (USA). River Systems 19/2:143-163.
5. Berry, C. R. Jr., M. Wildhaber, and D. L. Galat. 2004. Fish distribution and abundance. Volume 3 of Population structure and habitat use of benthic fishes along the Missouri and lower Yellowstone rivers. U.S. Geological Survey, Cooperative Fishery Units, South Dakota State University, Brookings, South Dakota.
6. ERB (USEPA Environmental Review Board). 2006. Environmental Administrative Decisions 12:490-707
7. Galat, D. L., C. R. Berry, W. M. gardner, J. C. Hendrickson, G. M. Mestl, G. J. Power, C. Stone, and M. R. Winston. 2005. American Fisheries Society Symposium 45:249-291.
8. Gibson, M.R. 1996. Comparison of trends in the finfish assemblage of Mt. Hope Bay and Narragansett Bay in relation to operations at the New England Power Brayton Point Station. RI Division Fish and Wildlife Research Reference Document 95/1. Revised August 1996.
9. Ohio Environmental Protection Agency (OEPA). 1978. Guidelines for the submittal of demonstrations pursuant to Sections 316(a) and 316(b) of the Clean Water Act and Chapter 3745-1 of the Ohio Administrative Code. Division of Industrial Wastewater, State of Ohio Environmental Protection Agency.
10. TVA (Tennessee Valley Authority). 2016. Biological monitoring of the Cumberland River near Cumberland Fossil Plant discharge during 2015. Knoxville, Tennessee.
11. Wilson, J.L., C. C. Coutant, and J. Tyner. 2014. Canton Mill Balanced and Indigenous Species Study of the Pigeon River (Clean Water Act Section 316(a) Demonstration). University of Tennessee, Knoxville, for Blue Ridge Paper Products, Inc. dba Evergreen Packaging, Canton Office, Canton, NC.
